# Constitutive GLI1 expression in chondrosarcoma is regulated by major vault protein via mTOR/S6K1 signaling cascade

**DOI:** 10.1038/s41418-021-00749-4

**Published:** 2021-02-26

**Authors:** Wei Wang, Taiqiang Yan, Wei Guo, Jianfang Niu, Zhiqing Zhao, Kunkun Sun, Hongliang Zhang, Yiyang Yu, Tingting Ren

**Affiliations:** 1 Musculoskeletal Tumor Center, Peking University People’s Hospital, Beijing, China; 2 Beijing Key Laboratory of Musculoskeletal Tumor, Beijing, China; 3 Department of Pathology, Peking University People’s Hospital, Beijing, China

**Keywords:** Transcription factors, Sarcoma, Protein-protein interaction networks, Oncogenes, Preclinical research

## Abstract

Hedgehog signaling plays a pivotal role in embryonic pattern formation and diverse aspects of the postnatal biological process. Perturbation of the hedgehog pathway and overexpression of GLI1, a downstream transcription factor in the hedgehog pathway, are highly relevant to several malignancies including chondrosarcoma (CS). We previously found that knocking down expression of GLI1 attenuates the disrupted Indian hedgehog (IHH) signal pathway and suppresses cell survival in human CS cells. However, the underlying mechanisms regulating the expression of GLI1 are still unknown. Here, we demonstrated the implication of GLI1 in SMO-independent pathways in CS cells. A GLI1 binding protein, major vault protein (MVP), was identified using the affinity purification method. MVP promoted the nuclear transport and stabilization of GLI1 by compromising the binding affinity of GLI1 with suppressor of fused homolog (SUFU) and increased GLI1 expression via mTOR/S6K1 signaling cascade. Functionally, knockdown of MVP suppressed cell growth and induced apoptosis. Simultaneous inhibition of MVP and GLI1 strongly inhibits the growth of CS in vitro and in vivo. Moreover, IHC results showed that MVP, GLI1, and P-p70S6K1 were highly expressed and positively correlated with each other in 71 human CS tissues. Overall, our findings revealed a novel regulating mechanism for HH-independent GLI1 expression and provide a rationale for combination therapy in patients with advanced CS.

## Introduction

Chondrosarcoma (CS) is a heterogeneous type of primary cartilage malignancy characterized by the accumulation of neoplastic chondrogenic cells [1]. Approximately 85% of CS cases are of the conventional primary type. Currently, surgical resection remains the standard intervention for this malignancy. However, the challenge of poor prognosis in patients still exists due to the limited role of chemotherapy and radiotherapy [2, 3]. Despite the numerous clinical trials on patients with advanced CS over the past few years [3–6], no standardized non-surgical option has been established. Therefore, there is an urgent need to identify novel treatment targets and develop effective strategies for combination therapy.

Hedgehog (HH) signaling plays a vital role in embryonic development and postnatal pattern formation including cell determination, specification, and stem cell renewal [7]. There are three types of HH ligands namely: sonic hedgehog (SHH), desert hedgehog (DHH), and Indian hedgehog (IHH) which exclusively regulates human skeletal development [7–9]. HH signaling is activated when HH ligands bind to and inactivate the patched receptor (PTCH). The smoothened (SMO) transmembrane protein is subsequently released to trigger downstream events, including disassociation of zinc finger protein GLIs from the suppressor of fused homolog (SUFU) and eventually expression of downstream target genes such as *GLI1*, *PTCH1*, and *HHIP* [7, 10]. There are three zinc finger GLI transcription factors in vertebrates. Deletion analysis in cultured cells revealed that GLI1 possesses only a C-terminal transcription activation domain thus primarily functions as a transcriptional activator, while GLI2 and GLI3 can either serve as an activator or repressor depending on post-translational modification [11, 12].

Biological and preclinical studies indicate that deregulation of IHH signaling largely contributes to the development of CS [3, 4, 9, 13]. However, direct inhibition of the upstream membrane receptor SMO is ineffective, which is evident from two clinical trials where SMO antagonists (IPI-926 and GDC-0449) were orally administered to patients with advanced CS. The patients either showed no improvement in PFS or failed to meet the primarily defined clinical benefit rate of 40% [5, 6]. Recently, the application of GLI1 to overcome anti-SMO resistance in several cancers has spurred great interest [14]. Our previous work showed that blocking the expression of GLI1 was an ideal strategy for suppressing the progression of CS [15]. However, compelling evidence suggests that post-translational modification of GLI1 and nonclassical mechanisms that are apparently independent of HH signaling also regulate the expression of the gene [11]. This GLI1 regulation system independent of the HH-PTCH1-SMO axis indicates the existence of an interface between HH signaling and other pathways during tumor progression. Therefore, it may present an opportunity to develop novel therapeutic targets for tumor conditions with aberrant expression of GLI1. Unfortunately, the mechanisms underlying the regulation of GLI1 expression in CS are still unclear.

Major vault protein (MVP) is the principal component of vaults, which are the largest ribonucleoprotein particles with hollow barrel-like structures [16]. MVP was found to be identical to the lung resistance-related protein (LRP) which is specifically over-expressed in multi-drug resistance cases of human lung cancer [17, 18]. Several proteins are reported to interact with MVP, including the estrogen receptor, Src, SHP2, COP1, and Shc3 [19–23]. Moreover, MVP is involved in pathways related to tumor development and multi-drug resistance, such as PI3K/AKT, MAPK/ERK, and the Notch signaling pathways [23–25]. However, the role of MVP in the development of CS and its impact on GLI1 or the IHH pathway is still unknown.

In this study, we established a novel interaction between GLI1 and MVP, and further illustrated their mechanism of action and functions at cellular and tissue levels. Our work confirms that MVP is a novel therapeutic target that suppresses GLI1 expression and the progression of CS. This advances our mechanistic understanding of HH independent regulation of GLI1 and provides a rationale for using combination therapy in CS.

## Materials and methods

### Human tissues

Thirteen fresh tissue samples (three articular cartilage, two osteochondroma, and eight conventional CS tissues), 71 paraffin-embedded conventional CS samples (31 Grade I, 24 Grade II, 16 Grade III tissues), and five paraffin-embedded articular cartilage tissues were collected from patients who underwent adequate tumor surgical resection at Musculoskeletal Tumor Center, Peking University People’s Hospital (Beijing, China). Clinical and histopathologic information for patients were collected from their medical records.

### Cell lines and reagents

Four human CS cell lines SW1353 (Grade II), OUMS-27 (Grade III), HCS-2/8 (well-differentiated), CS-OKB (Grade II) were used in this study [26–28]. SW1353 was purchased from American type culture collection (ATCC HTB-94; Manassas, VA, USA). OUMS-27, HCS-2/8, and CS-OKB were kindly granted by Dr. J Block (Rush Medical College, Chicago, IL, USA). SW1353 was authenticated by BGI Co., LTD (Beijing, China) with short tandem repeat analysis, the results were compared with the ATCC and DSMZ databases, other cell lines were not registered in these databases. SW1353 was maintained with L-15, whereas OUMS-27, HCS-2/8, CS-OKB were cultured in DMEM supplemented with 10% FBS and 1% antibiotics. The primary chondrocyte was isolated with collagenase II and grown in DMEM/F12, and certificated with cartilage-specific genes (type II, X collagen, and aggrecan) expression by RT-PCR. HEK-293T cell line was purchased from ATCC (ACS-4500). Following reagents were purchased from MedChemExpress (Monmouth, NJ, USA): cyclopamine (HY-17024), cycloheximide (CHX) (HY-12320), MG-132 (HY-13259), PF-04691502 (HY-15177), GANT61 (HY-13901); human recombinant IHH (#78197) was purchased from STEMCELL Technologies (Vancouver, Canada).

### Plasmids, siRNA, and transfection

Lentiviral vectors that express SMO, MVP, and 3xFlag tagged human full-length GLI1 were constructed using the vector GV492 (Genechem lnc., Shanghai, China). Empty lentiviral vectors (LV-vector) were used as a negative control. Recombinant plasmids encoding GFP tagged human full-length MVP, 3xFlag tagged truncated GLI1, V5 tagged human full-length SUFU, and lentiviral vectors that encode predesigned short hairpin RNAs (shRNAs) targeting MVP and GLI1 were obtained from Likely Biotechnology (Beijing, China). Recombinant plasmids coding shRNAs against p70 S6K1 were purchased from OriGene Technologies (TF320520). Stable gene over-expression or knock-down cell clones were generated by lentivirus infection following by 3 mg/ml puromycin selection for 1 week. Duplexes of siRNA that target MVP (siMVP), SMO (siSMO), and non-target scramble siRNAs were designed and synthesized by GenePharma (Suzhou, Jiangsu, China). Transient siRNA transfection was performed using Lipofectamine 3000 Transfection Reagent (L3000015, Thermo Scientific). Details on the shRNA targeted sequence and siRNA duplexes sequence were provided (Supplementary Table 3).

### Immunoprecipitation (IP) and western blotting (WB)

Cells were collected and lysed with cell lysis buffer (#9803, CST) supplemented with protease and phosphatase inhibitor cocktail (78440, Thermo Scientific) for 30 min. The protein solution was collected and pretreated with protein A/G PLUS-Agarose (sc-2003, Santa Cruz Biotechnology) for 30 min to decrease non-specific binding. The whole-cell lysate protein dilution was incubated with primary antibody overnight at 4 °C. Protein A/G PLUS-agarose was added and co-incubated at 4 °C for another 1.5 h. The pellet was collected after centrifugation, samples were boiled for 10 min after the addition of 40 μl 3 × loading buffer (#7722, CST). For WB, proteins were separated by SDS-PAGE gel electrophoresis and wet-transferred onto PVDF membrane. After blocking with Tris-buffered saline containing 5% skim milk and 0.1% Tween 20, the membrane was incubated together with diluted primary antibody at 4 °C overnight. The membrane was washed and incubated with the corresponding HRP-conjugated secondary antibody for 1 h at 37 °C. Protein bands were visualized with the Bio-Rad ChemiDOC™ XRS Imaging system. Details of the primary antibodies used for IP and WB are listed in Supplementary Table 4.

### Chromatin immunoprecipitation (CHIP)

CHIP was performed using the CHIP-IT Express kit(53008, Active Motif) following the manufacturer’s protocol. Briefly, 1.5 × 10^7^ cells were fixed with 37% formaldehyde (252549, Sigma Aldrich) to crosslink and preserve protein–DNA interactions. The samples were then sheared on ice using a Covaris sonicator until 200–1200 bp DNA fragments were obtained. Protein-bound DNA sequences were immunoprecipitated using ChIP-grade antibodies against GLI1 (NB600-600, Novus Biologicals) and a negative control IgG (#2729, CST). Protein was digested using proteinase K and crosslinking was reversed using NaCl. The purified DNA was collected and subjected to standard PCR techniques. Primers used for PCR amplification were: PTCH1 promoter, forward 5′–GAGCATTCCTTAATGGAAG–3′, reverse 5′–CTGCAACGCGATTGGCTCT–3′; GAPDH control: forward 5′–GAAGGTCGGAGTCAACGGATTT–3′, reverse 5′–ATGGGTGGAATCATATTGGAAC–3′.

### Dual-luciferase reporter assay

Luciferase reporter plasmids containing four repeats of the GLI protein DNA binding sequences 5′–GACCACCCAC–3′ were constructed. For luciferase assay, cells were plated in a 12-well plate and transfected with 1 μg luciferase reporter plasmids and 500 ng of pRL-TK plasmids (E2231, Promega Madison, WI) using Lipofectamine 3000. Forty-eight hours after transfection and treatment, cells were then lysed and subjected to the luciferase activity analysis using the Dual-Luciferase Assay Kit (E1910, Promega Madison, WI) according to the manufacturer’s instructions. All luciferase activities were normalized for transfection efficiency according to pRL-TK luciferase activity.

### Immunohistochemistry (IHC) and Immunofluorescence (IF)

The procedure for IHC was the same as our previous study [15]. The staining scores were assessed by two independent pathologists without any previous information on the clinical specimens. Briefly, the staining results were quantified by staining intensity and percentage of positive cells. Staining intensity was categorized and recorded as 0, 1, 2, 3, representing negative, low, moderate, and strong staining, respectively. Percentage of positive cells was calculated and recorded as 0, 1, 2, 3, 4, which indicates positive staining cells accounts for 0–5%, 5–25%, 25–50%, 50–75%, >75 of total cell numbers. Staining intensity multiplied by the percentage of positive cells is the weighted score for each area, the average score of 5 random areas is the final staining score for each case. Grading was based on the average scores: 0–1 (−); 1–4 (+); 4–8 (++); >8 (+++). For IF cells were seeded in 24 well plates and treated with an indicated agent, then fixed cells were permeabilized with 0.1% Triton X-100 (T8200, Solarbio Life Sciences) for 15 min, after blocking with normal goat serum, cells were incubated with primary antibody at 4 °C overnight. The next day, cells were washed with PBS with 0.1% Tween 20 (PBST) and incubated with CoraLite488 or CoraLite594 conjugated secondary antibody at 37 °C for 1 h. DAPI was used to stain the nucleus. The samples were then analyzed with confocal microscopy (Leica TCS-SP8, Germany). Primary antibodies used for IHC and IF are described in Supplementary Table 4.

### Cell proliferation assays

Cells were plated in 96 well plates at a density of 5000 cells per well. After overnight culture, the medium was replaced with the complete medium that was either drug-free or contained treatment agents. Cells were cultured for the indicated time after which cell viability was measured by CCK-8 (Dojindo Laboratories, Kumamoto, Japan) according to the manufacture’s protocol. To perform growth curve assay, cells were plated in 6-well plates at an initial density of 10^4^ cells per well. At each time point, cells were counted and re-planked with the same cell numbers. For colony formation assay, 800 cells were uniformly plated in a 6-well plate, the medium was replaced every 3 days. After 2–3 weeks of incubation, fixed cell colonies were stained with 0.1% crystal violet (G1063, Solarbio Life Sciences) and counted.

### Flow cytometry

SW1353 and CS-OKB cells were transfected with scrambled siRNA or siMVP for 48 h, after which cells were digested with EDTA-free trypsin. After washing trice with PBS, cells were incubated with Annexin V/PI kit (BD Biosciences, San Jose, CA, USA) in darkness according to the manufacture’s protocol and analyzed with a flow cytometer. Cells that stained positive for Annexin V and PI were recognized as late apoptosis.

### RNA-Sequencing and RT-PCR

Total RNA was extracted with Trizol reagent (15596018, Invitrogen) as the manufacture’s instruction. RNA purity and concentration were measured using Nanophotometer Pearl (IMPLEN, CA, USA). Preparation of RNA library and transcriptome sequencing was conducted by Novogene Co., LTD (Beijing, China). Genes with adjusted *p*-value < 0.05 and |log2(FoldChange)| > 0 were considered as differentially expressed. For RT-PCR, 1 μg total RNA was converted to complementary DNA (cDNA) using PrimeScriptTM RT reagent Kit (DRR037A, TaKaRa, Dalian, Liaoning, China) in a volume of 20 μl. The real-time PCR assay was conducted in a CFX96 real-time PCR detection system (Bio-Rad, CA, USA). GAPDH served as the internal control. Primer sequences are listed in Supplementary Table 3.

### Tumorigenicity assay and combination therapy in vivo

Four-week-old female BALB/c nude mice were housed in SPF condition. Animals were randomly allocated into four groups (*n* = 4, one mouse died during the process of drug treatment in the GANT61 single-agent group). For tumorigenesis, 5 × 10^6^ HCS2/8 or HCS2/8-shMVP cells were injected subcutaneously into the right flank of the mice. The tumor volumes were calculated using the formula: *I* × *W*^2^, with *I* denoting the longest tumor diameter and *W* is the shortest. For combination therapy, 5 × 10^6^ HCS2/8-shMVP cells were subcutaneously injected into the right flanks of the mice and allowed to grow for 7 days, GANT61 (40 mg/kg), dissolved in corn oil (HY-Y1888, MCE, NJ, USA) were subcutaneously injected and the tumor volume was measured with calipers every 3 days. Tumor weight and tumor volume data are presented as mean ± SD.

### Statistics

The density of the western blot band was measured by Image J software. Differences between the two groups were assessed by one-way Student’s *t*-test, Pearson chi-square test, and Fisher’s exact test were used to analyzing the correlation of protein expression. Statistical analysis was performed with SPSS 22.0 software and *p*-value < 0.05 was considered statistically significant. Biological experiments were repeated at least three independent times.

### Study approval

All animal experiments were approved by the Institutional Review Board of Peking University, People’s Hospital and carefully conducted according to Guide for the Care and Use of Laboratory Animals. The specimen collection was conducted according to the protocols approved by the ethics committee of Peking University People’s Hospital and informed consent was obtained from patients in all cases.

## Results

### GLI1 expression is not affected by SMO inhibition in CS cell line SW1353

**Fig. 1:**
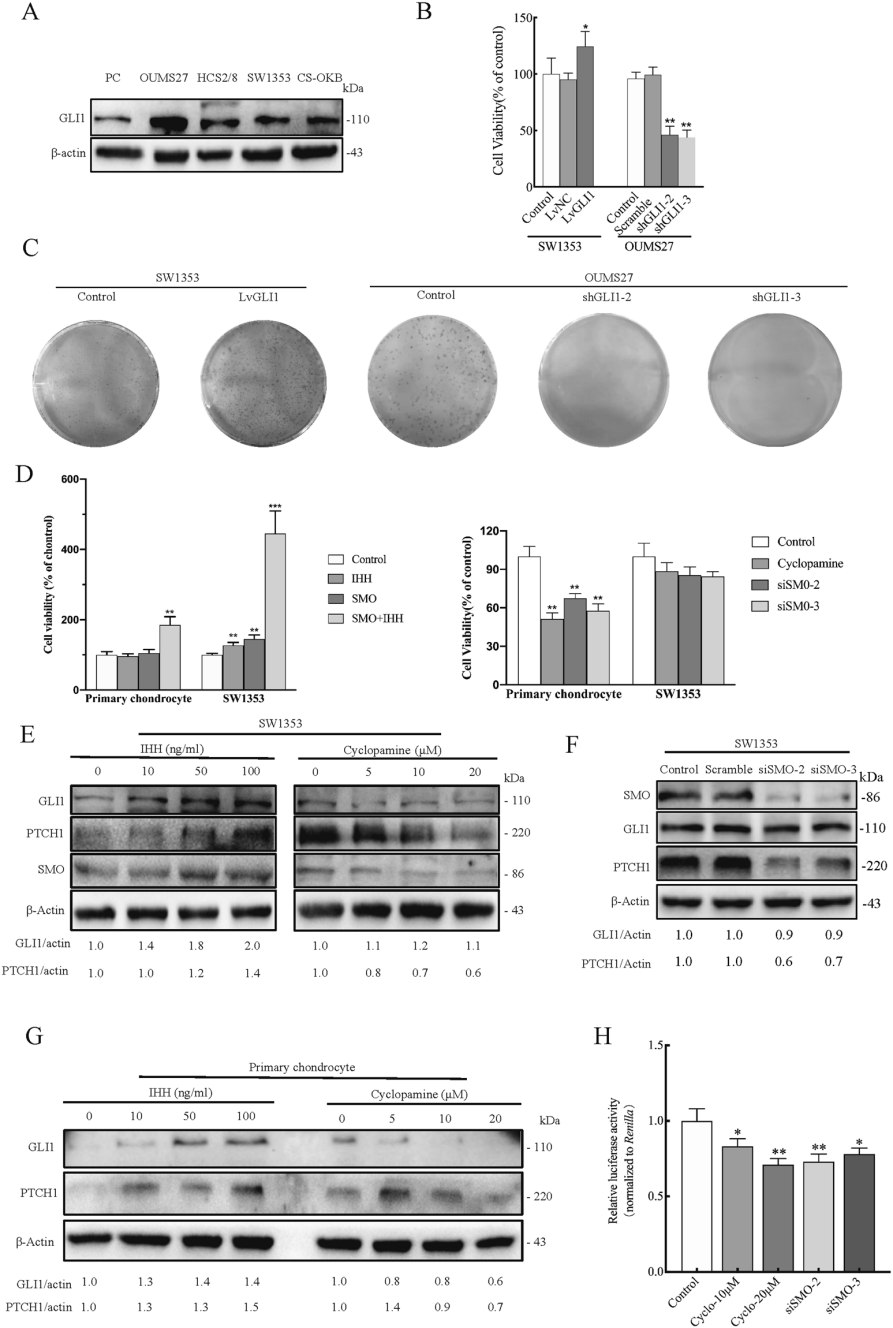
The expression of GLI1 is not affected by SMO inhibition in CS. **A** Western blot analysis of GLI1 expression in primary chondrocytes (PC) and four CS cell lines. **B** Cell viability was measured using the CCK-8 assay in CS cells over-expressing GLI1 or GLI1 knockdown CS cells. Error bars represent SD (*n* = 6). **C** A colony formation assay was performed to evaluate the impact of GLI1 on the proliferation of CS cells. **D** Cell viability was measured using the CCK-8 assay after the treatment of IHH pathway activation and SMO inhibition in primary chondrocyte and SW1353. Recombinant Human IHH (100 ng/ml) and cyclopamine (20 μM) were treated for 24 h. Error bars represent SD (*n* = 6). **E** Western blot analysis of hedgehog pathway components in SW1353 cells after 24 h treatment with IHH or cyclopamine. β-Actin served as the loading control. The blots between IHH and cyclopamine were conditioned at different exposure times to achieve optimal saturation. Densitometry was performed for quantification and the relative ratios of GLI1 and PTCH1 were shown below the blots. **F** Western blot analysis of hedgehog pathway components in SW1353 after SMO knockdown. **G** Western blot analysis of GLI1 and PTCH1 in primary chondrocyte after 24 h treatment with IHH or cyclopamine. Given that GLI1/PTCH1 expression is undetectable in primary chondrocytes, IHH (50 ng/ml) was added 12 h before cyclopamine treatment to stimulate the background expression of GLI1 and PTCH1. β-Actin served as the loading control. **H** Relative luciferase activity of the GLIBS-Luc reporter in SW1353 after SMO inhibition. Cyclopamine was treated for 24 h. Error bars represent SD (*n* = 4). All data are presented as the mean ± SD (*p < 0.05, **p < 0.01,***p < 0.001, by Student’s *t*-test).

First, four CS cell lines and primary chondrocytes were compared based on GLI1 expression. The results indicated that GLI1 was over-expressed in the CS cell lines. The highest expression was observed in OUMS27 while the other three CS cell lines showed the same level of expression (Fig. 1A). Moreover, exogenous expression of GLI1 in SW1353 increased cell viability and ability to form clones. However, knockdown of GLI1 in OUMS27 significantly impaired cell proliferation (Fig. 1B, C). These results, therefore, confirm that GLI1 is essential for cell proliferation in CS.

SMO was then activated or inhibited in primary chondrocytes and SW1353 (Supplementary Fig. 1A). This was done to evaluate whether GLI1 expression was regulated by the canonical IHH/SMO/GLI1 axis in CS cells. Interestingly, SW1353 displayed increased sensitivity to exogenous IHH stimulation but less sensitivity to SMO inhibition. When SMO over-expressed cells were treated with 100 ng/ml IHH, the viability of the SW1353 cells increased about 3.5-fold, while that of the primary chondrocytes increased by a mere 0.85-fold. On the contrary, the viability of the SW1353 cells slightly decreased after SMO inhibition while the rate of inhibition in primary chondrocytes was noted at about 60% (Fig. 1D; Supplementary Fig. 8A).

Furthermore, protein abundance analysis revealed that PTCH1 was regulated by the IHH ligand and cyclopamine in SW1353 cells. Interestingly, despite GLI1 expression being up-regulated by IHH and SMO over-expression, its expression was not apparently affected by inhibition of SMO (Fig. 1E, F; Supplementary Fig. 1B). There was, however, a remarkable decrease in the expression of GLI1 in primary chondrocytes after treatment with cyclopamine (Fig. 1G). We then employed a GLI-dependent luciferase reporter system (GLIBS-Luc) to characterize the transcriptional activity of GLIs and CHIP-PCR to specifically determine the occupancy of GLI1 on its target gene. From the results, although SMO inhibition could decrease the recruitment of GLI1 on *PTCH1-*promoter, its suppressive activity on GLIBS-Luc reporter was modest (Fig. 1H; Supplementary Fig. 1C).

Taken together, these results indicate that knockdown of SMO is insufficient to suppress the expression of GLI1 and HH pathway activity in SW1353, suggesting the presence of pathways orthogonal to the IHH pathway that causes SMO-independent activation of GLI1.

### GLI1 forms complex with MVP and mTOR through the domain of SUFU binding sites

**Fig. 2:**
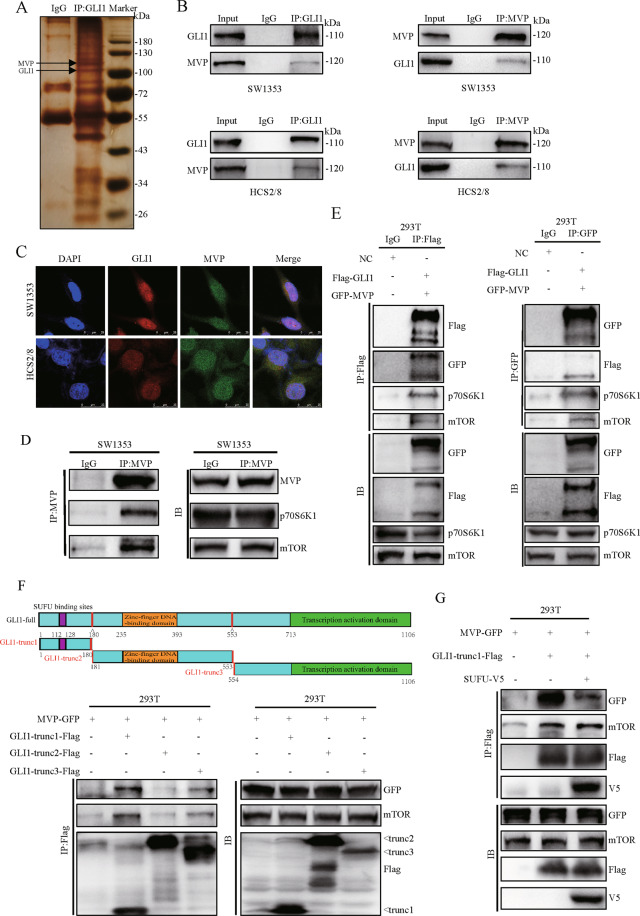
GLI1 forms complex with MVP and mTOR through the region of SUFU binding sites. **A** Silver staining results of IP with GLI1 antibody in SW1353 cell lysates. Normal IgG was used as the negative control. **B** SW1353 and HCS2/8 cells were lysed and subjected to IP-western blot assay to determine the interaction between endogenous GLI1 and MVP. **C** Immunofluorescence analysis of GLI1 and MVP distribution in CS cell lines. DAPI was used to stain nuclei. **D** The interaction between MVP and mTOR pathway components was examined using IP with MVP antibody. **E** HEK-293T cells were transiently transfected with empty vector or Flag-GLI1 together with GFP-MVP, cells were lysed and subjected to IP-western blot assay 48 h after transfection to examine the interaction of GLI1, MVP, mTOR, and p70S6K1. **F** HEK-293T cells were transiently transfected with MVP-GFP, GLI1-trunc1-Flag (SUFU binding sites), GLI1-trunc2-Flag (zinc-finger DNA-binding domain), and GLI1-trunc3-Flag (transcription activation domain). Cells were harvested 48 h after transfection followed by IP-western blot assay. **G** HEK-293T cells were transiently transfected with MVP-GFP, GLI1-trunc1-Flag, and SUFU-V5. Cells were harvested 48 h after transfection followed by IP-western blot assay.

Analysis of proteins that interact with GLI1 is useful in identifying pathways that regulate GLI1’s function. In this case, IP combined with LC-MS/MS was used to determine proteins that bind with GLI1. Mass spectrometry results showed that GLI1 interacts with HSP60, MVP, eEF1A2 et al., these protein interactions were further verified by western blot (Supplementary Fig. 2). Silver staining indicated that MVP is associated with GLI1 (Fig. 2A). IP using GLI1 antibody pulled down MVP. On the contrary, IP using MVP antibody pulled down GLI1, no protein band was detected in the IgG control group (Fig. 2B). IF results showed that both GLI1 and MVP were co-localized in the nucleus (Fig. 2C), this suggested a functional correlation between the two proteins.

Activation of the mTOR/S6K1 pathway contributes to the development and progression of CS [3]. Studies have reported that MVP has an effect on the activation of the mTOR pathway [24, 25]. However, the mechanisms linking MVP, GLI1, and mTOR/S6K1 signaling in CS are not clear. Our study here found that MVP interacts with mTOR and its downstream kinase p70S6K1 in SW1353 (Fig. 2D). When plasmids that express Flag-tagged GLI1 and GFP tagged MVP were transfected into HEK-293T cells, IP with Flag antibody not only pulled down MVP but also p70S6K1 and mTOR. On the other hand, IP with GFP antibody not only pulled down GLI1 but also p70S6K1 and mTOR (Fig. 2E). In summary, these data indicate that GLI1, MVP, p70S6K1, and mTOR form a protein complex in CS cells.

The functional domains for GLI1 include SUFU bindings sites, DNA binding domain, and C-terminal transcriptional activation domain [11, 29, 30]. To map the specific domain of GLI1 necessary for its interaction with mTOR and MVP, plasmids that express Flag-tagged truncated GLI1 fragments of different functional domains were co-transfected with GFP tagged MVP into HEK-293T. Results showed that the truncated fragment containing SUFU binding sites has the most potent binding force with MVP and mTOR (Fig. 2F). It was concluded that the domain containing SUFU binding sites enhances GLI1 to form a complex with MVP and mTOR.

To investigate whether MVP and SUFU bound to GLI1 competitively, plasmids expressing V5 tagged SUFU were co-transfected with GLI1-trunc1-flag and MVP-GFP into HEK-293T. Results showed that MVP interacted with the truncated GLI1 fragment containing SUFU binding sites. However, ectopic expression of SUFU impaired this interaction but did not suppress the association between GLI1-trunc1-Flag and mTOR (Fig. 2G). We then knocked down MVP in stable exogenous Flag-tagged GLI1 over-expressed SW1353 cells. IP with Flag antibody pulled down more SUFU but did not affect the interaction between GLI1 and mTOR (Supplementary Fig. 3A). In contrast, over-expression of MVP reduced the interaction between GLI1 and SUFU (Supplementary Fig. 3B). These results suggest that MVP impairs binding between GLI1 and SUFU, which is independent of the mTOR pathway.

### MVP promotes the nuclear localization and protein stabilization of GLI1

**Fig. 3:**
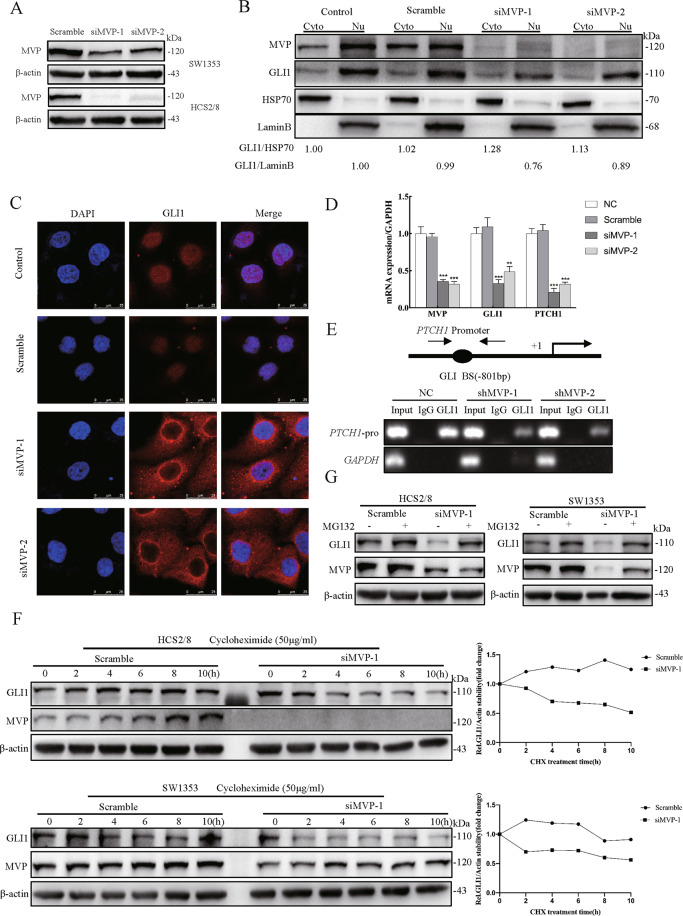
MVP facilitates GLI1 nuclear localization and stabilization. **A** SW1353 and HCS2/8 cells were transiently transfected with scrambled siRNA and siMVP for 48 h, the knockdown efficiency was determined using western blot. **B** Western blot analysis of GLI1 distribution after transient knockdown of MVP followed by nuclear-cytosolic protein isolation in HCS2/8. HSP70 and Lamin B were used as cytoplasmic and nuclei markers, respectively. The relative expression ratio of GLI1 was shown below the blot. **C** Immunofluorescence analysis of GLI1 distribution in HCS2/8 after transient transfection with siMVP for 48 h, non-specific target scramble siRNA was used as the control. **D** Real-time RT-PCR results showing GLI1 target gene expression after MVP knockdown in HCS2/8. Error bars represent SD (*n* = 6). **E** Stable shMVP HCS2/8 cell clones were constructed, and chromatin was immunoprecipitated with the antibody of GLI1 and isotope control IgG. Eluted DNA was PCR-amplified using primers encompassing the GLI binding site of the *PTCH1-*promoter or the *GAPDH* coding region. **F** SW1353 and HCS2/8 cells were treated with cycloheximide (CHX, 50 μg/ml) for the indicated times, and cell lysates were analyzed with western blot with the indicated antibody. β-actin was used as the loading control, quantification results are shown in the right panel. **G** SW1353 and HCS2/8 cells were pre-treated with DMSO or MG132 (10 μM) for 8 h, followed by parallel co-treatment with siMVP for 24 h. Cells were lysed and analyzed by western blot with indicated antibodies. All data are presented as the mean ± SD (*p < 0.05, **p < 0.01,***p < 0.001, by Student’s t-test).

During mammalian HH signaling transduction, SUFU functions to sequester GLI1 in the cytoplasm and promote the degradation of GLI1 through the ubiquitin-proteasome system [29, 31]. Since MVP impairs GLI1’s binding force with SUFU, there is speculation that the nuclear transport and protein stability of GLI1 is affected by MVP. Firstly, when the efficiency of MVP knockdown was compared in SW1353 and HCS2/8, the latter with higher knockdown efficiency was selected for subsequent research (Fig. 3A). WB results showed that GLI1 abundance decreased in the nucleus but increased in the cytoplasm after MVP knock-down (Fig. 3B). IF results showed that GLI1 was highly concentrated within the cytoplasm of HCS2/8-siMVP cells, with a ring-shaped high expression region around the nucleus (Fig. 3C). In addition, the knockdown of MVP significantly downregulated the recruitment of GLI1 on the *PTCH1* promoter and mRNA expression of *PTCH1* and *GLI1* (Fig. 3D, E). These data indicate that MVP plays an essential role for the nuclear transportation and target genes transcription of GLI1 in CS cells.

Protein stability assay was then performed by blocking endogenous protein synthesis with CHX. Approximately one-half of the endogenous GLI1 was degraded within 10 h in two MVP-knockdown cell lines (Fig. 3F; Supplementary Fig. 4A). On the other hand, expression of GLI1 in untreated cell lines was stable and exhibited a slight decrease in protein expression within 10 h. Similarly, GLI1 abundance decreased with MVP knock-down but could be restored through pharmacological blockade of the proteasome activity with MG132 (Fig. 3G; Supplementary Fig. 4B). The data above suggest that MVP promotes nuclear localization and protein stability of GLI1 in CS cell lines.

### MVP promotes GLI1 expression by activating the mTOR/S6K1 pathway

**Fig. 4:**
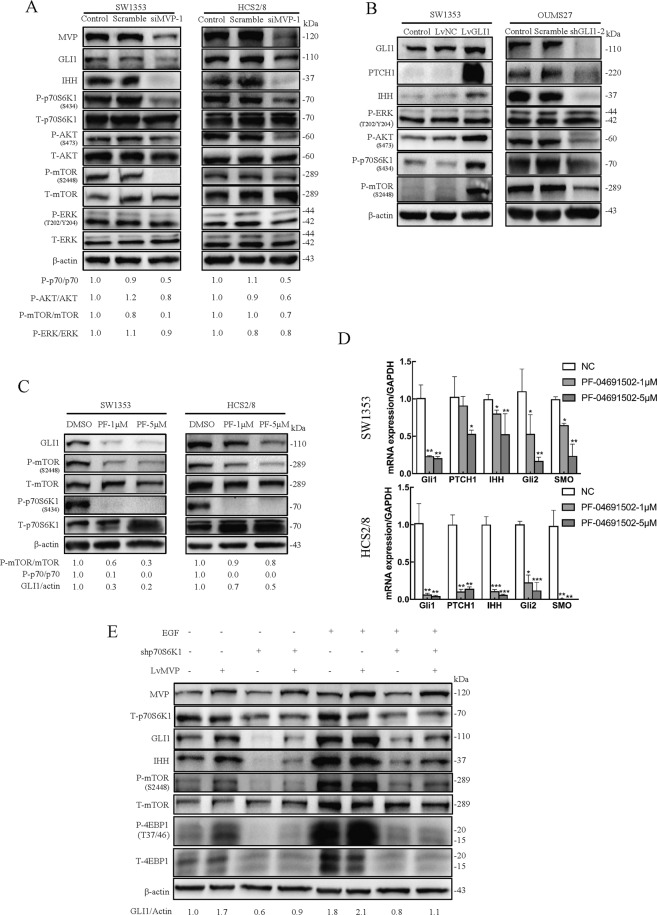
MVP promotes GLI1 expression by activating the mTOR/S6K1 signaling cascade. **A** Western blot analysis of principle proteins involved in IHH, mTOR, and MAPK signaling pathway in control and CS cells with MVP knock-down. Densitometry analysis was performed to quantify the relative phosphorylation levels of p70S6K1, AKT, mTOR, and ERK based on the blots. **B** Western blot results showing the expression of indicated proteins in SW1353 cells over-expressing GLI1 and OUMS27 cells with GLI1 knockdown. **C** SW1353 and HCS2/8 were treated with dual PI3K/mTOR inhibitor PF-04691502(PF) for 24 h, cell lysates were subjected to western blot analysis with the indicated antibody. β-actin served as the loading control and the relative expression of GLI1 and phosphorylated p70S6K1 and mTOR are shown below the blots. **D** Real-time RT-PCR results showing the expression of genes related to the IHH pathway after treatment with PF-04691502 for 24 h.Error bars represent SD (*n* = 6). **E** Western blot analysis showing expression of proteins associated with IHH and mTOR pathway in HCS2/8 cells over-expressing MVP transfected with shp70S6K1 in the presence or absence of 10 ng/ml EGF. All data are presented as the mean ± SD (*p < 0.05, **p < 0.01,***p < 0.001, by Student’s *t*-test).

The role of MVP in regulating nuclear localization and stability of GLI1 and formation of a protein complex with GLI1 and mTOR/S6K1 is a suggestion that MVP, GLI1, and mTOR/S6K1 are functionally related. First, while investigating the effect of MVP on the IHH and mTOR pathway, MVP in HCS2/8 and SW1353 cell lines were knocked down, respectively. It was determined that the knockdown of MVP attenuated not only GLI1 and IHH expression, but also the elevated HH pathway activity by exogenous IHH stimulation (Fig. 4A, Supplementary Fig. 5A–C). On the other hand, MVP knockdown lowered the phosphorylation levels of mTOR pathway components but showed no positive results on the MAPK pathway (Fig. 4A; Supplementary Fig. 5A). The data above suggest that MVP, as a crucial regulator, enhances the activation of IHH and mTOR pathways in CS.

The role of GLI1 in activating IHH and mTOR pathway was then explored. Stable over expression of GLI1 (Supplementary Fig. 8B) in SW1353 leads to increased expression of PTCH1 and IHH and increased phosphorylation levels of mTOR pathway components. Reciprocally, when GLI1 was knocked down (Supplementary Fig. 8C) in OUMS27, reduced expression of PTCH1 and IHH and phosphorylation levels of mTOR pathway members were observed, while ERK was not affected (Fig. 4B; Supplementary Fig. 5D–F). Moreover, since SW1353 showed higher phosphorylation levels of AKT and p70S6K1, while the phosphorylation levels in OUMS27 were relatively low (Fig. 7B), GLI1 was overexpressed in OUMS27 and knocked down in SW1353. Results showed that the phosphorylation levels of mTOR pathway members increased after GLI1 was overexpressed and decreased after GLI1 knockdown (Supplementary Fig. 5G, H). These data indicate that when GLI1 is over-expressed in CS cells, activation of IHH and mTOR pathways occur.

To investigate the functions of mTOR pathway on GLI1 expression, SW1353 and HCS2/8 were treated with the PI3K/mTOR dual inhibitor PF-04691502. Results showed that PF-04691502 significantly inhibited 80% (SW1353) and 50% (HCS2/8) GLI1 expression at 5 μM concentration (Fig. 4C). Using RT-PCR, it was shown that the expression of other IHH pathway members (PTCH1, IHH, GLI2, and SMO) significantly reduced after PF-04691502 treatment (Fig. 4D). These results therefore suggest that GLI1 over-expression activates the mTOR pathway, while mTOR pathway inhibition inversely reduces the expression of GLI1.

Based on the results above, we speculated that the mTOR pathway partially is involved in regulating GLI1 expression, enhanced by MVP within the CS cells. Since MVP exhibited low expression in HCS2/8 (Fig. 7B), we validated the speculation by examining GLI1 expression in stable MVP over-expressed HCS2/8 cell line followed by p70S6K1 knock-down. Results revealed that MVP increased the expression of GLI1, which was significantly enhanced when stimulated with EGF. When p70S6K1 was subjected to a knockdown and visualized at low phosphorylation levels of 4E-BP1 or mTOR, GLI1 abundance decreased and a reduction in over-expressed MVP induced elevated GLI1 expression was observed. Knockdown of p70S6K1 directly inhibited GLI1 expression at elevated levels, which was stimulated by EGF (Fig. 4E). These results suggest that GLI1 expression is partially regulated by MVP through activating the mTOR/S6K1 cascade.

### MVP promotes CS cell proliferation and prevents apoptosis

**Fig. 5:**
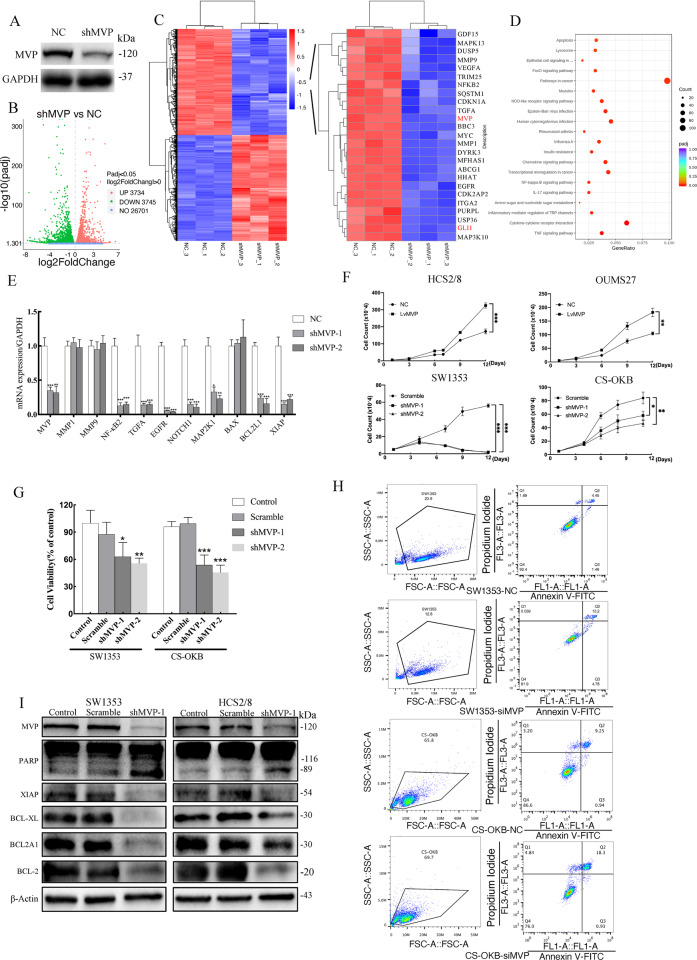
MVP promotes proliferation and reduces cell apoptosis in CS cell lines. **A** Stable shMVP SW1353 cell clone was constructed. **B** Volcano plots showing the number of differentially expressed genes between shMVP transfected cells and the control group. **C** Heat map showing differentially expressed genes between shMVP transfected cells and control group, several down-regulated genes are listed in the right panel, including MVP and GLI1. Each sample was analyzed in triplicate. **D** KEGG analysis of the down-regulated genes after MVP knockdown. **E** RT-PCR on SW1353-shMVP cells was performed to validate the results from the RNA-seq. Error bars represent SD (*n* = 6). **F** Growth curves of CS cells over-expressing MVP or with MVP-knockdown. Error bars represent SD (*n* = 3). **G** Cell viability of clones with stable MVP knockdown was measured using CCK-8 assay. Non-target shRNA was used as a negative control. Error bars represent SD (*n* = 6). **H** Apoptotic cells were detected using the Annexin V/PI kit and results were analyzed by flow cytometry in siMVP and scramble NC group. **I** Western blot analysis of apoptosis-related proteins in shMVP transfected and control cells. All data are presented as mean ± SD (*p < 0.05, **p < 0.01,***p < 0.001, by Student’s *t*-test).

Activation of IHH and mTOR pathways enhances malignant progression in CS [13, 32]. The role of MVP in activating these two pathways indicate that MVP is linked to CS development. To investigate the functions of MVP, a stable SW1353-shMVP cell line was constructed (Fig. 5A). RNA-sequencing analysis revealed that 3734 genes were significantly up-regulated while 3745 genes were down-regulated (Fig. 5B). Among the down-regulated genes, GLI1 and some other tumor progression related genes were discovered, for example, *MMP1*, *MMP9*, *NFKB2*, *TGFA,* and *EGFR* (Fig. 5C). Analysis using KEGG suggested that the down-regulated genes were highly enriched in “Pathways in Cancer” with the largest gene count numbers (Fig. 5D). Drawing insights into the functions of these genes, several tumor proliferation-related genes (*EGFR*, *NOTCH1*, and *MAP2K1*) and the anti-apoptosis related genes (*BCL2L1*, *XIAP*) were detected (Supplementary Table 1). Validation with qPCR revealed that expression of *MVP, NFKB2, TGFA, EGFR, NOTCH1, MAP2K1, BCL2L1*, and *XIAP* were suppressed in SW1353-shMVP cells except for *MMP1, MMP9*, and *BAX*, whose expression had no significant difference with the NC group (Fig. 5E). This indicated that MVP may be involved in regulating CS proliferation and apoptosis.

To confirm this hypothesis, MVP was over-expressed in the low MVP expression cell lines HCS2/8 and OUMS27 and knocked down in SW1353 and CS-OKB (Supplementary Fig. 8D–G) with relatively high MVP expression. It was observed that the presence of MVP significantly promoted tumor cell proliferation (Fig. 5F, G; Supplementary Fig. 6). Flow cytometry revealed that a proportion of late-phase apoptotic cells in SW1353 increased by 8.55%, CS-OKB increased by 9.05% after the knock-down of MVP by siRNA (Fig. 5H). In addition, the anti-apoptotic proteins XIAP, BCL-xl, BCL2A1, BCL-2 were reduced, and PARP was cleaved after MVP knockdown (Fig. 5I; Supplementary Fig. 7). From the above results, it was indicated that MVP functions to promote tumor cell proliferation and inhibit apoptosis in CS.

### Combined inhibition of MVP and GLI1 in CS provides better therapeutic effects

**Fig. 6:**
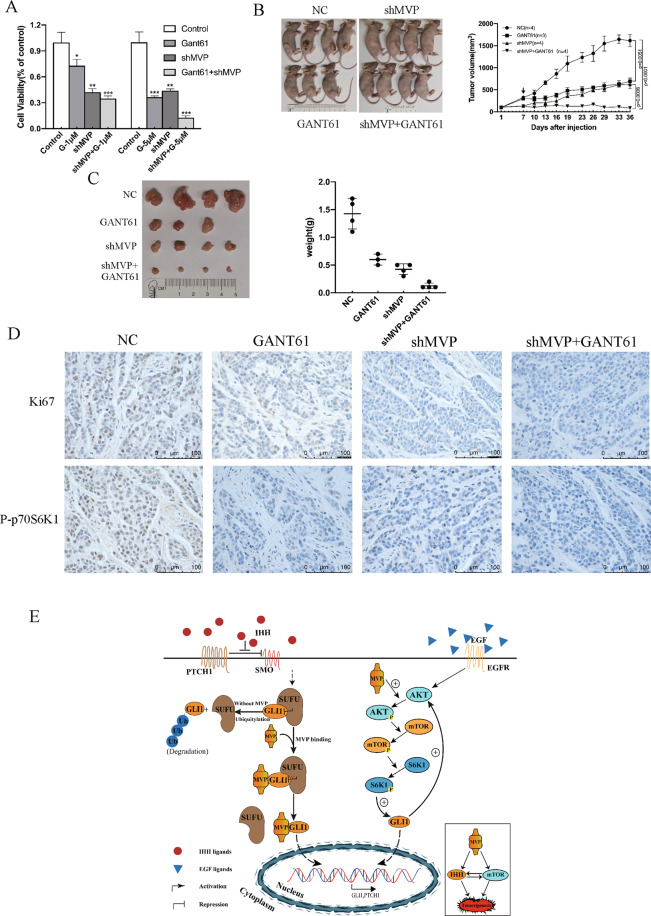
Simultaneous inhibition of MVP and GLI1 strongly inhibits the growth of CS. **A** Cell viability of SW1353 after treatment with GANT61 or/and shMVP. Error bars represent SD (*n* = 6). **B** In vivo combination therapy for subcutaneously inoculated tumors using shMVP or/and GANT61 (40 mg/kg). In total, 5 × 10^6^ HCS2/8 cells or HCS2/8 clones stably transfected with shMVP were subcutaneously injected into the right flank of nude mice, tumor cells were allowed to grow for 7 days before GANT61 or the solvent (corn oil) were administered. GANT61 was subcutaneously injected and the tumor volumes were measured and calculated every 3 days. Error bars represent SD (*n* = 4, the GANT61 single-agent group *n* = 3). **C** Images of tumors from the indicated groups and weights of resected tumors. Error bars represent SD (*n* = 4, the GANT61 single-agent group *n* = 3). **D** Representative IHC images of Ki67 and P-p70S6K1 in resected tumors. **E** Schematic representation of MVP-mediated GLI1 stabilization, nuclear localization and activation.

Over-expression of GLI1 is known to promote CS progression. This work therefore, has shown that presence of MVP in CS cells is necessary for GLI1 expression, MVP knockdown thus may enhance the killing effects of GLI1 inhibitor. Based on the results, cell viability was reduced to 36% with 5 μM GANT61 treatment in SW1353 and was about 44% after MVP knockdown. The addition of 5 μM GANT61 in stable shMVP cells reduced cell activity to about 13% (Fig. 6A).

To investigate the combined therapeutic effects in vivo, HCS2/8 and HCS2/8-shMVP cells were subcutaneously injected into the nude mice. Knockdown of MVP or treatment with GANT61 significantly inhibited tumor growth, however, combined inhibition of MVP and GLI1 showed additive synergistic anti-tumor effect (Fig. 6B, C). When the resected tumors were subjected to IHC analysis, GANT61 and shMVP reduced P-p70S6K1 and Ki67 expression. The combined inhibition indicated synergistic inhibitory effects (Fig. 6D). These results suggest that the use of a combination therapy that targets both MVP and GLI1 provides better therapeutic effects.

### GLI1, MVP, and P-p70S6K1 are positively correlated in human CS tissues

**Fig. 7:**
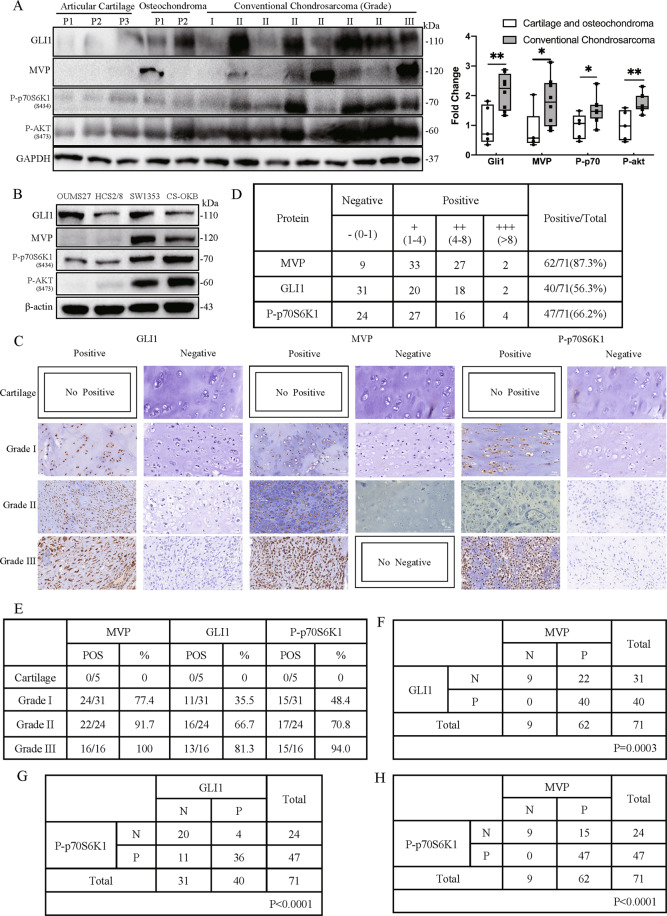
GLI1, MVP and P-p70S6K1 expression are positively correlated in human CS tissues. **A** Western blot results showing expression of GLI1, MVP, P-p70S6K1, and P-AKT in tissues from normal articular cartilage, osteochondroma, and conventional CS. Densitometry was performed to quantify the relative expression of these proteins as shown in the right box plot. **B** Western blot results showing expression of GLI1, MVP, P-p70S6K1, and P-AKT in four CS cell lines. **C** Representative images of positive or negative IHC staining results of GLI1, MVP, and P-p70S6K1. **D** Statistical analysis of IHC staining results from 71 human conventional CS tissues. **E** Statistical analysis of positive IHC staining results for MVP, GLI1, and P-p70S6K1 in five normal cartilage and 71 conventional CS tissues. **F** Statistical analysis showing correlation between GLI1 and MVP in IHC staining results of 71 human conventional CS tissues. **G** Statistical analysis for the correlation between P-p70S6K1 and GLI1 based on IHC staining results of 71 human conventional CS tissues. **H** Statistical analysis for the correlation between P-p70S6K1 and MVP based on IHC staining results of 71 human conventional CS tissues.

We then explored the expression of MVP, GLI1, and P-p70S6K1 in CS tissues. Western blot results showed that expression of GLI1, MVP, P-p70S6K1, and P-AKT were enhanced in conventional CS as compared to the expression in non-malignant normal cartilage and osteochondroma (Fig. 7A). Interestingly, MVP was found to have high correlation with P-p70S6K1 and P-AKT expression in 4 CS cell lines (Fig. 7B). We further confirmed the correlation among MVP, GLI1 and P-p70S6K1 by examining the expression of the three proteins in 71 cases of conventional CS and five cases of normal articular cartilage. Out of the 71 cases, 62, 40, 47 positive cases were for MVP, GLI1 and P-p70S6K1 respectively. No positive staining results were detected in five articular cartilages (Fig. 7C, D). Further analysis showed that an increase in histological grade leads to a gradual increase in the positive protein expression rates (Fig. 7E), but the expression of these proteins did not correlate with gender, age and location of the tumors (Supplementary Table 2). Moreover, strong correlations between MVP and GLI1, MVP and P-p70S6K1, GLI1 and P-p70S6K1 were observed (Fig. 7F–H). These results confirmed that MVP, GLI1, and mTOR pathways are aberrantly activated in CS and justified the existence of a functional correlation between them.

## Discussion

Our previous study demonstrated that the aberrantly activated IHH signaling pathway facilitates CS development and progression. Blocking of the transcription factor GLI1 at the distal end of IHH pathway is a promising strategy for remedying CS [13, 15]. This study further validates the effects of blocking transcriptional activity of GLI1 in animal models, revealing a novel regulation mechanism for GLI1 expression in CS (Fig. 6E). This provides a novel therapeutic target for CS treatment and urgent the necessary for combination therapy.

The canonical IHH signaling pathway has a tight negative feedback loop which inhibits SMO activation by promoting the expression of PTHrP and PTCH1 [7, 9, 33]. Mutations within the components of the HH pathway, for example, membrane receptor proteins PTCH1 and SMO [34, 35], non-membrane receptor proteins, such as SUFU [36, 37] and REN (KCTD11) [38], disrupt the feedback loop causing activation of HH pathway and tumor progression. We previously established that HH pathway activation in CS is hardly caused by PTCH1 and SMO mutations [39]. Genome-wide screening has revealed that low frequencies of mutations of IHH pathway components occur in conventional CS [40]. Therefore, activation of the IHH pathway may attribute to downstream lesions or sensitivity to IHH pathway regulators. This research indicates that CS cells are more sensitive to IHH ligand than the primary chondrocytes, and treatment with a single SMO inhibitor rarely hampers cell viability. Despite GLI1 being crucial in maintaining cell survival, its expression is not attenuated by SMO inhibitor. These findings confirm the crucial role of GLI1 in IHH pathway activation and indicates that over-expression of GLI1 in CS is regulated via SMO-independent pathways.

SMO-independent activation of GLI1 has been detected in several malignant tumors such as esophageal adenocarcinoma whereby activated S6K1 phosphorylates GLI1 at Ser84 to induce GLI1 transcriptional activity and oncogenic functions [41]. Similarly, IKBKE directly promotes nuclear localization and transcriptional activity of GLI1 in pancreatic ductal adenocarcinoma [42]. In the present study, MVP, as a more potent upstream regulator than SMO, promoted the nuclear transport and stabilization of GLI1 by compromising the binding affinity of GLI1 with SUFU and increased GLI1 expression via mTOR/S6K1 signaling cascade. It indicates that while IHH may signal through SMO to activate GLI1, it is the non-canonical activation of GLI1 that dominates in CS cells, and this proposed novel regulatory mechanism of MVP/mTOR/GLI1 axis may possibly explain the failure of recent clinical trials using SMO antagonists in patients with advanced CS.

In addition, reports have indicated that MVP is over-expressed in several types of tumors, and is associated with MDR phenotype, cell survival and migration [18–25, 43]. Present study indicated that MVP was positively expressed in 87.3% of human CS tissue samples. RNA-sequencing displayed a decrease in genes associated with tumorigenesis and progression with the knockdown of MVP. MVP is identified as a novel molecule that is essential in the progression of CS therefore, blocking MVP expression acts as an ideal strategy for treating CS. In addition, as evidenced to be identical to LRP, MVP contributes to chemotherapy failure in several malignant tumors such as lung cancer, breast cancer, and hepatocellular carcinoma [17, 23, 24]. Further in vitro and in vivo studies are required to determine whether MVP causes chemotherapy resistance in CS.

IF and IHC analysis demonstrated that MVP in CS cell lines and tissue samples are predominantly concentrated in the nucleus and not in the cytoplasm like most eukaryotic cells [44, 45]. MVP may have different cellular distribution and functions in different cell types. For example, high expression of MVP within the nucleus in CS indicates its tendency to influence gene transcription by regulating transcription factors. In addition, it was observed that the expression of MVP, GLI1, and P-p70S6K1 has a positive correlation within human CS tissues. Strikingly, there was no positive expression of GLI1 or P-p70S6K1 based on MVP negative expression out of the 71 conventional CS samples. This confirmed the vital regulatory role of MVP in GLI1 and P-p70S6K1 activation during CS development. Moreover, MVP, GLI1 and P-p70S6K1 frequently exhibit significant co-expression suggesting that most patients may experience simultaneous activation of MVP, IHH and mTOR pathways. Such patients may not benefit from a single antagonist, but rather from combined simultaneous inhibition of two or three pathways. It is worth noting that small molecule inhibitors targeting members of GLI1 and mTOR pathway are currently being developed [46–48], and are increasingly being proven as effective inhibitors applied in clinical trials. Exploration of multi-target combination treatment strategy may provide more effective treatment options for CS.

In summary, our results revealed a novel interaction between GLI1 and MVP, and established a crosstalk of GLI1 with MVP/mTOR/S6K1 pathways thereby providing a novel regulation mechanism for SMO-independent GLI1 expression. The present research also revealed that MVP, as a crucial GLI1 regulator enhances proliferation to tumor cells and anti-apoptosis process. Combined inhibition of GLI1 and MVP enhances therapeutic effects in vitro and in vivo. Moreover, a positive correlation between GLI1, MVP and P-p70S6K1 indicates that they are functionally relevant, and provides a rationale for combined targeted therapy.

## Supplementary information

Supplementary Tables

Supplementary Figure 1

Supplementary Figure 2

Supplementary Figure 3

Supplementary Figure 4

Supplementary Figure 5

Supplementary Figure 6

Supplementary Figure 7

Supplementary Figure 8

Supplementary information
