# Supplementary material for: Constitutive GLI1 expression in chondrosarcoma is regulated by major vault protein via mTOR/S6K1 signaling cascade
Source: Cell Death Differ. 2021 Feb 26;28(7):2221–37. doi: 10.1038/s41418-021-00749-4 (PMC8257592; doi:10.1038/s41418-021-00749-4)
Supplement: Supplementary file 1 — Supplementary Tables [file 41418_2021_749_MOESM1_ESM.docx]

**Supplementary Tables**

Constitutive GLI1 expression in chondrosarcoma is regulated by major vault protein via mTOR/S6K1 signaling cascade

Wei Wang, Taiqiang Yan,^*^ Wei Guo, ^*^ Jianfang Niu, Zhiqing Zhao, Kunkun Sun, Hongliang Zhang, Yiyang Yu, Tingting Ren

**Supplementary Table 1.**

**Differentially expressed genes in “Pathways in cancer" of KEGG analysis**

| **gene_name** | **Log2FoldChange** | **padj** | **gene_description** |
| --- | --- | --- | --- |
| LAMB3 | -2.082705 | 0 | laminin subunit beta 3 |
| CXCL8 | -7.5278958 | 7.07E-131 | C-X-C motif chemokine ligand 8 |
| HSP90B1 | -1.2483779 | 3.15E-123 | heat shock protein 90 beta family member 1 |
| MMP9 | -3.3107208 | 2.40E-101 | matrix metallopeptidase 9 |
| VEGFA | -1.4912223 | 3.11E-92 | vascular endothelial growth factor A |
| NFKB2 | -1.4747873 | 2.18E-82 | nuclear factor kappa B subunit 2 |
| TRAF1 | -3.2098903 | 5.55E-70 | TNF receptor associated factor 1 |
| CDKN1A | -0.8562917 | 8.64E-57 | cyclin dependent kinase inhibitor 1A |
| BIRC3 | -3.7540252 | 4.94E-56 | baculoviral IAP repeat containing 3 |
| TGFA | -1.8785277 | 1.15E-41 | transforming growth factor alpha |
| PLCG2 | -1.1837094 | 2.11E-35 | phospholipase C gamma 2 |
| EPAS1 | -0.8425987 | 1.05E-34 | endothelial PAS domain protein 1 |
| BBC3 | -1.3744443 | 2.81E-34 | BCL2 binding component 3 |
| MYC | -0.8349105 | 6.78E-34 | MYC proto-oncogene, bHLH transcription factor |
| MMP1 | -2.4094696 | 9.41E-34 | matrix metallopeptidase 1 |
| IL2RB | -2.1448916 | 6.11E-33 | interleukin 2 receptor subunit beta |
| LAMC3 | -1.7919881 | 1.70E-30 | laminin subunit gamma 3 |
| GADD45A | -0.8368032 | 3.61E-30 | growth arrest and DNA damage inducible alpha |
| SLC2A1 | -0.7286068 | 1.39E-29 | solute carrier family 2 member 1 |
| NFKBIA | -1.1163365 | 8.12E-29 | NFKB inhibitor alpha |
| EGFR | -0.7788338 | 1.55E-22 | epidermal growth factor receptor |
| BAX | -0.7107888 | 5.21E-22 | BCL2 associated X, apoptosis regulator |
| DVL1 | -0.5872102 | 3.36E-21 | dishevelled segment polarity protein 1 |
| TRAF3 | -0.6426281 | 3.74E-20 | TNF receptor associated factor 3 |
| LAMC2 | -2.4043035 | 1.73E-19 | laminin subunit gamma 2 |
| ITGA2 | -0.9053725 | 3.04E-19 | integrin subunit alpha 2 |
| RARA | -0.8530675 | 3.42E-17 | retinoic acid receptor alpha |
| ADCY3 | -0.6790298 | 5.34E-17 | adenylate cyclase 3 |
| GSTO1 | -0.6135757 | 3.46E-16 | glutathione S-transferase omega 1 |
| IL4R | -0.6373523 | 1.43E-14 | interleukin 4 receptor |
| PIM1 | -0.7183832 | 1.76E-14 | Pim-1 proto-oncogene, serine/threonine kinase |
| HEY1 | -1.4833582 | 3.64E-13 | hes related family bHLH transcription factor with YRPW motif 1 |
| MDM2 | -0.5490121 | 7.36E-13 | MDM2 proto-oncogene |
| PRKCB | -2.3775846 | 2.44E-12 | protein kinase C beta |
| AXIN1 | -0.5544816 | 5.35E-11 | axin 1 |
| BRAF | -0.5940978 | 1.15E-10 | B-Raf proto-oncogene, serine/threonine kinase |
| GLI1 | -1.1876198 | 1.39E-10 | GLI family zinc finger 1 |
| GNA12 | -0.3568932 | 6.51E-10 | G protein subunit alpha 12 |
| RAC2 | -0.5479393 | 6.84E-10 | Rac family small GTPase 2 |
| PPARG | -0.9672704 | 7.29E-10 | peroxisome proliferator activated receptor gamma |
| JAG1 | -0.658266 | 1.16E-09 | jagged 1 |
| EGLN1 | -0.5011784 | 3.82E-09 | egl-9 family hypoxia inducible factor 1 |
| NOTCH1 | -0.5545403 | 4.86E-09 | notch 1 |
| MAX | -0.5050205 | 4.91E-09 | MYC associated factor X |
| IL6 | -7.7375127 | 5.72E-09 | interleukin 6 |
| RALA | -0.3621107 | 1.01E-08 | RAS like proto-oncogene A |
| IL15RA | -1.1763497 | 2.72E-08 | interleukin 15 receptor subunit alpha |
| LAMA1 | -0.5499898 | 4.42E-08 | laminin subunit alpha 1 |
| IL23A | -2.3094274 | 1.27E-07 | interleukin 23 subunit alpha |
| BCL2L1 | -0.4247452 | 1.54E-07 | BCL2 like 1 |
| IFNGR1 | -0.5604489 | 2.74E-07 | interferon gamma receptor 1 |
| CTBP2 | -0.3605953 | 2.75E-07 | C-terminal binding protein 2 |
| CAMK2G | -0.4670392 | 5.55E-07 | calcium/calmodulin dependent protein kinase II gamma |
| TGFBR1 | -0.3897571 | 7.65E-07 | transforming growth factor beta receptor 1 |
| ETS1 | -0.3847225 | 1.28E-06 | ETS proto-oncogene 1, transcription factor |
| TRAF5 | -1.0342008 | 1.63E-06 | TNF receptor associated factor 5 |
| BCL2L11 | -0.8080145 | 2.19E-06 | BCL2 like 11 |
| EPOR | -0.8916878 | 2.75E-06 | erythropoietin receptor |
| HES1 | -0.9634454 | 3.68E-06 | hes family bHLH transcription factor 1 |
| MAP2K1 | -0.3760096 | 3.72E-06 | mitogen-activated protein kinase kinase 1 |
| VEGFB | -0.4017384 | 9.86E-06 | vascular endothelial growth factor B |
| GSTO2 | -0.9856297 | 1.16E-05 | glutathione S-transferase omega 2 |
| DVL2 | -0.3881438 | 1.46E-05 | dishevelled segment polarity protein 2 |
| CCND1 | -0.2656276 | 1.72E-05 | cyclin D1 |
| PIM2 | -0.8721469 | 1.88E-05 | Pim-2 proto-oncogene, serine/threonine kinase |
| GSTP1 | -0.2583594 | 2.06E-05 | glutathione S-transferase pi 1 |
| PLCG1 | -0.318124 | 2.95E-05 | phospholipase C gamma 1 |
| RUNX1 | -0.3576965 | 4.54E-05 | runt related transcription factor 1 |
| CASP7 | -0.4873201 | 0.00010372 | caspase 7 |
| VHL | -0.2939044 | 0.00015472 | von Hippel-Lindau tumor suppressor |
| FGF19 | -4.1786818 | 0.00020904 | fibroblast growth factor 19 |
| CBL | -0.3617703 | 0.00022153 | Cbl proto-oncogene |
| GNB5 | -0.3734263 | 0.00027891 | G protein subunit beta 5 |
| BDKRB2 | -0.8922846 | 0.00032979 | bradykinin receptor B2 |
| NOTCH2 | -0.3429511 | 0.00033942 | notch 2 |
| IL6ST | -0.306533 | 0.00036643 | interleukin 6 signal transducer |
| PTGER4 | -0.6546311 | 0.0003992 | prostaglandin E receptor 4 |
| ADCY2 | -1.2584832 | 0.00043356 | adenylate cyclase 2 |
| CSF2RA | -1.2072886 | 0.00050461 | colony stimulating factor 2 receptor alpha subunit |
| FZD3 | -0.5272885 | 0.00055594 | frizzled class receptor 3 |
| ADCY1 | -1.0457346 | 0.00071962 | adenylate cyclase 1 |
| TRAF2 | -0.4206348 | 0.00076958 | TNF receptor associated factor 2 |
| MET | -0.296184 | 0.00080145 | MET proto-oncogene, receptor tyrosine kinase |
| HMOX1 | -0.2119557 | 0.0008585 | heme oxygenase 1 |
| WNT7A | -0.7510072 | 0.00086756 | Wnt family member 7A |
| DDB2 | -0.392405 | 0.00110295 | damage specific DNA binding protein 2 |
| RAC3 | -1.0295962 | 0.00111615 | Rac family small GTPase 3 |
| FGFR4 | -1.4065715 | 0.0011776 | fibroblast growth factor receptor 4 |
| EGLN3 | -0.4512754 | 0.00138922 | egl-9 family hypoxia inducible factor 3 |
| RPS6KA5 | -0.7833877 | 0.00139274 | ribosomal protein S6 kinase A5 |
| IL15 | -0.6550701 | 0.00193751 | interleukin 15 |
| ADCY6 | -0.2422194 | 0.00253497 | adenylate cyclase 6 |
| JAK2 | -0.4107045 | 0.00327423 | Janus kinase 2 |
| IL2RG | -1.1185225 | 0.00430221 | interleukin 2 receptor subunit gamma |
| ADCY7 | -0.314644 | 0.00471461 | adenylate cyclase 7 |
| CYCS | -0.1957564 | 0.00477472 | cytochrome c, somatic |
| ARAF | -0.212179 | 0.00731827 | A-Raf proto-oncogene, serine/threonine kinase |
| GNG7 | -0.4808565 | 0.00778312 | G protein subunit gamma 7 |
| LAMB4 | -2.6499865 | 0.00848603 | laminin subunit beta 4 |
| ITGA2B | -1.453416 | 0.01033363 | integrin subunit alpha 2b |
| NTRK1 | -2.0362784 | 0.01045532 | neurotrophic receptor tyrosine kinase 1 |
| FZD4 | -0.3631772 | 0.0110847 | frizzled class receptor 4 |
| ELK1 | -0.2500007 | 0.01124485 | ELK1, ETS transcription factor |
| SMAD2 | -0.2096578 | 0.01130064 | SMAD family member 2 |
| IL12RB1 | -1.3804437 | 0.01854033 | interleukin 12 receptor subunit beta 1 |
| ZBTB17 | -0.2866042 | 0.01967551 | zinc finger and BTB domain containing 17 |
| FOS | -1.1623028 | 0.02638251 | Fos proto-oncogene, AP-1 transcription factor subunit |
| XIAP | -0.1676113 | 0.02738216 | X-linked inhibitor of apoptosis |
| CALM3 | -0.1522606 | 0.03083344 | calmodulin 3 |
| GNB3 | -0.5790875 | 0.03359776 | G protein subunit beta 3 |
| CKS1B | -0.2511496 | 0.03454872 | CDC28 protein kinase regulatory subunit 1B |
| CEBPA | -0.5481268 | 0.03492203 | CCAAT enhancer binding protein alpha |
| FOXO1 | -0.3753301 | 0.03573451 | forkhead box O1 |
| BID | -0.2834955 | 0.04376406 | BH3 interacting domain death agonist |

**Supplementary Table 2.**

**Correlation between the exression of GLI1, MVP, P-p70S6K1 and the clinical characters in 71 CS patients**

|  | Number(n) | GLI1 | | *P* value | MVP | | *P* value | P-p70S6K1 | | *P* value |
| --- | --- | --- | --- | --- | --- | --- | --- | --- | --- | --- |
|  |  | Pos^†^ | Pos(%) |  | Pos | Pos(%) |  | Pos | Pos(%) |  |
| Gender |  |  |  |  |  |  |  |  |  |  |
| Male | 42 | 23 | 55 | 0.747 | 39 | 93 | 0.146 | 29 | 69 | 0.541 |
| Female | 29 | 17 | 59 |  | 23 | 79 |  | 18 | 62 |  |
| Age |  |  |  |  |  |  |  |  |  |  |
| <30 | 12 | 5 | 42 | 0.719 | 9 | 75 | 0.5571 | 7 | 67 | 0.685 |
| 30-49 | 9 | 5 | 56 |  | 8 | 89 |  | 5 | 56 |  |
| 50-60 | 37 | 22 | 59 |  | 33 | 89 |  | 25 | 68 |  |
| >60 | 13 | 8 | 62 |  | 12 | 92 |  | 10 | 77 |  |
| Location |  |  |  |  |  |  |  |  |  |  |
| Spine***^a^*** | 14 | 7 | 50 | 0.864 | 12 | 86 | 0.131 | 10 | 71 | 0.280 |
| Girdle***^b^*** | 36 | 21 | 58 |  | 34 | 94 |  | 26 | 72 |  |
| Limb | 21 | 12 | 57 |  | 16 | 76 |  | 11 | 52 |  |

Spine^a^: including Sacrum、Thoracic and Lumbar Vertebra and Chest Wall.

Girdle^b^: including Hip、Scapula and Clavicle.

Pos^†^: number of positive specimens.

**Supplementary Table 3.**

**The shRNA target sequences, siRNA sequences and Primer sequences**

| **shRNA** | **Target Sequence(5'-3')** |
| --- | --- |
| **Scramble** | TCCTAAGGTTAAGTCGCCCTC |
| **GLI1-1** | GAGCCTGAATCTGTGTATGAA |
| **GLI1-2** | CTGCAGTAAAGCCTTCAGCAA |
| **GLI1-3** | AGCCGAGTATCCAGGATACAA |
| **MVP-1** | ACATGGATATAGTGGTATGGG |
| **MVP-2** | CCCATCAACCTCTTCAACACA |

| **siRNA duplex** | **Sense(5'-3')** | **Antisense(5'-3')** |
| --- | --- | --- |
| **Scramble** | UUCUCCGAACGUGUCACGUTT | ACGUGACACGUUCGGA GAATT |
| **MVP-1** | GCAACUGAAGAGUUCAUCATT | UGAUGAACUCUUCAGUUGCTT |
| **MVP-2** | GGAGGUCGUGGAGAUCAUUTT | AAUGAUCUCCACGACCUCCTT |
| **SMO-1** | GGCUACAAGAACUACCGAUTT | AUCGGUAGUUCUUGUAGCCTT |
| **SMO-2** | GCUUUGUGCUCAUUACCUUTT | AAGGUAAUGAGCACAAAGCTT |
| **SMO-3** | CCAUGUUUGGAACUGGCAUTT | AUGCCAGUUCCAAACAUGGTT |

| **Primer for RT-PCR** | **Forward(5'-3')** | **Reverse(5'-3')** |
| --- | --- | --- |
| **GLI1** | GAGAAGCCGAGCCGAGTAT | TGGTGAGTAGACAGAGGTTGG |
| **MVP** | CTGAGGTGGAGGTGAAGAAGTT | GCTGTGTTGAAGAGGTTGATGG |
| **PTCH1** | TGTGATGGATGCTGGAACCT | CCTACATAATGCCTGCCTCTAC |
| **IHH** | ACTTCTGCCTGGTCCTGTTG | CTGTGTTCTCCTCGTCCTTGA |
| **GLI2** | CCATCTCCACGACTACCTCAA | CTCACTGCTCTGCTTGTTCTG |
| **SMO** | TCTGTCCTGCGTCATCATCTT | CGGTATCGGTAGTTCTTGTAGC |
| **Type II Collagen** | CCTGGCAAAGATGGTGAGACAG | CCTGGTTTTCCACCTTCACCTG |
| **Type X Collagen** | CGCTGAACGATACCAAATGCCC | TGGACCAGGAGTACCTTGCTCT |
| **Aggrecan** | TACGACGCCATCTGCTACA | TCTCCAGTCTCATTCTCAACCT |
| **MMP1** | ATGAAGCAGCCCAGATGTGGAG | TGGTCCACATCTGCTCTTGGCA |
| **MMP9** | GCCACTACTGTGCCTTTGAGTC | CCCTCAGAGAATCGCCAGTACT |
| **NF-kB2** | GGCAGACCAGTGTCATTGAGCA | CAGCAGAAAGCTCACCACACTC |
| **TGFA** | GGTCCGAAAACACTGTGAGTGG | CAAACTCCTCCTCTGGGCTCTT |
| **EGFR** | CCAGGCTATCAATCAGGAAGAC | CCAGGCTATCAATCAGGAAGAC |
| **NOTCH1** | GGTGAACTGCTCTGAGGAGATC | GGATTGCAGTCGTCCACGTTGA |
| **MAP2K1** | GGTGTTCAAGGTCTCCCACAAG | CCACGATGTACGGAGAGTTGCA |
| **BAX** | TCAGGATGCGTCCACCAAGAAG | TGTGTCCACGGCGGCAATCATC |
| **BCL2A1** | GCCACTTACCTGAATGACCACC | AACCAGCGGTTGAAGCGTTCCT |
| **XIAP** | TGGCAGATTATGAAGCACGGATC | AGTTAGCCCTCCTCCACAGTGA |
| **GAPDH** | GAAGGTCGGAGTCAACGGATTT | ATGGGTGGAATCATATTGGAAC |

**Supplementary Table 4.**

**Details about the primary antibodies**

| **No.** | **Product Name** | **Product Number** | **Manufacture** | **Host and Clonality** | **Application** |
| --- | --- | --- | --- | --- | --- |
| **1** | Anti-Gli1 Antibody | ab217326 | Abcam | Anti Rabbit Polyclonal Antibody | WB,IHC |
| **2** | GLI1 (C68H3) Rabbit mAb | #3538 | Cell Signaling Technology | Anti Rabbit Monoclonal Antibody | IP |
| **3** | GLI1 (L42B10) Mouse mAb | #2643 | Cell Signaling Technology | Anti Mouse Monoclonal Antibody | IP |
| **4** | GLI1-Antibody | NBP1-78259 | Novus Biologicals | Anti Rabbit Polyclonal Antibody | WB,IF |
| **5** | MVP/LRP Antibody | 16478-1-AP | Proteintech | Anti Rabbit Polyclonal Antibody | WB,IP |
| **6** | LRP Antibody (1014) | sc-23916 | Santa Cruz Biotechnology | Anti Mouse Monoclonal Antibody | IP,IHC,IF |
| **7** | Ihh Antibody (H-88) | sc-13088 | Santa Cruz Biotechnology | Anti Rabbit Polyclonal Antibody | WB |
| **8** | PTCH1 (C53A3) Rabbit mAb | #2468 | Cell Signaling Technology | Anti Rabbit Monoclonal Antibody | WB |
| **9** | SMO Antibody | DF5152 | Affinity Biosciences | Anti Rabbit Polyclonal Antibody | WB |
| **10** | SUFU (C81H7) Rabbit mAb | #2522 | Cell Signaling Technology | Anti Rabbit Monoclonal Antibody | WB |
| **11** | p70 S6 Kinase Antibody | #9202 | Cell Signaling Technology | Anti Rabbit Polyclonal Antibody | WB |
| **12** | p-p70 S6 kinase α Antibody (A-6) | sc-8416 | Santa Cruz Biotechnology | Anti Mouse Monoclonal Antibody | WB,IHC |
| **13** | mTOR (7C10) Rabbit mAb | #2983 | Cell Signaling Technology | Anti Rabbit Monoclonal Antibody | WB |
| **14** | Phospho-mTOR (Ser2448) (D9C2) XP® Rabbit mAb | #5536 | Cell Signaling Technology | Anti Rabbit Monoclonal Antibody | WB |
| **15** | Phospho-Akt (Ser473) (D9E) XP® Rabbit mAb | #4060 | Cell Signaling Technology | Anti Rabbit Monoclonal Antibody | WB |
| **16** | Akt (pan) (11E7) Rabbit mAb | #4685 | Cell Signaling Technology | Anti Rabbit Monoclonal Antibody | WB |
| **17** | Phospho-p44/42 MAPK (Erk1/2) (Thr202/Tyr204) (D13.14.4E) XP® Rabbit mAb | #4370 | Cell Signaling Technology | Anti Rabbit Monoclonal Antibody | WB |
| **18** | p44/42 MAPK (Erk1/2) (137F5) Rabbit mAb | #4695 | Cell Signaling Technology | Anti Rabbit Monoclonal Antibody | WB |
| **19** | Phospho-4E-BP1 (Thr37/46) (236B4) Rabbit mAb | #2855 | Cell Signaling Technology | Anti Rabbit Monoclonal Antibody | WB |
| **20** | 4E-BP1 (53H11) Rabbit mAb | #9644 | Cell Signaling Technology | Anti Rabbit Monoclonal Antibody | WB |
| **21** | PARP Antibody | #9542 | Cell Signaling Technology | Anti Rabbit Polyclonal Antibody | WB |
| **22** | XIAP Antibody | 10037-1-Ig | Proteintech | Anti Rabbit Polyclonal Antibody | WB |
| **23** | Bcl-XL Antibody | 10783-1-AP | Proteintech | Anti Rabbit Polyclonal Antibody | WB |
| **24** | A1/Bfl-1 (D1A1C) Rabbit mAb | #14093 | Cell Signaling Technology | Anti Rabbit Monoclonal Antibody | WB |
| **25** | BCL2 Antibody | 12789-1-AP | Proteintech | Anti Rabbit Polyclonal Antibody | WB |
| **26** | HSP60 Antibody | 15282-1-AP | Proteintech | Anti Rabbit Polyclonal Antibody | WB |
| **27** | HSPA1L Antibody | 13970-1-AP | Proteintech | Anti Rabbit Polyclonal Antibody | WB |
| **28** | EEF1A2 Antibody | 16091-1-AP | Proteintech | Anti Rabbit Polyclonal Antibody | WB |
| **29** | RPS27A Antibody | 14946-1-AP | Proteintech | Anti Rabbit Polyclonal Antibody | WB |
| **30** | HSP70 (D69) Antibody | #4876 | Cell Signaling Technology | Anti Rabbit Polyclonal Antibody | WB |
| **31** | Lamin B1 Antibody | 12987-1-AP | Proteintech | Anti Rabbit Polyclonal Antibody | WB |
| **32** | Monoclonal ANTI-FLAG® M2 antibody produced in mouse | F1804 | Sigma Aldrich | Anti Mouse Monoclonal Antibody | WB,IP |
| **33** | V5 Tag Antibody | 14440-1-AP | Proteintech | Anti Rabblit Polyclonal Antibody | WB |
| **34** | GFP Tag Antibody | 66002-1-Ig | Proteintech | Anti Mouse Monoclonal Antibody | WB,IP |
| **35** | Mouse Anti-β actin mAb | TA-09 | ZSGB-BIO | Anti Mouse Monoclonal Antibody | WB |
| **36** | Mouse Anti-GAPDH mAb | TA-08 | ZSGB-BIO | Anti Mouse Monoclonal Antibody | WB |
