# Supplementary figures and images for: Constitutive GLI1 expression in chondrosarcoma is regulated by major vault protein via mTOR/S6K1 signaling cascade
Source: Cell Death Differ. 2021 Feb 26;28(7):2221–37. doi: 10.1038/s41418-021-00749-4 (PMC8257592; doi:10.1038/s41418-021-00749-4)

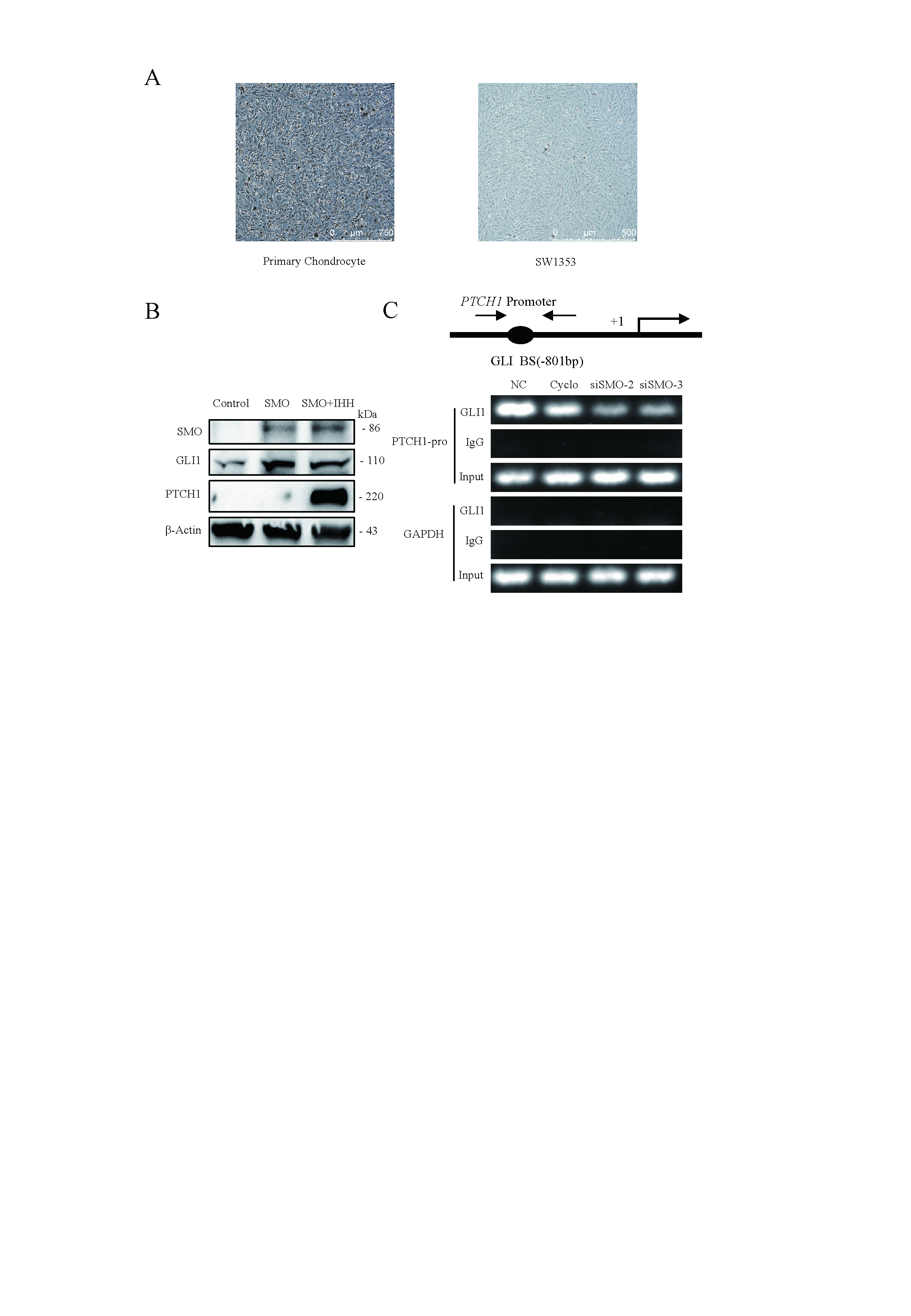

Supplement: Supplementary file 2 — Supplementary Figure 1 [file 41418_2021_749_MOESM2_ESM.tif]

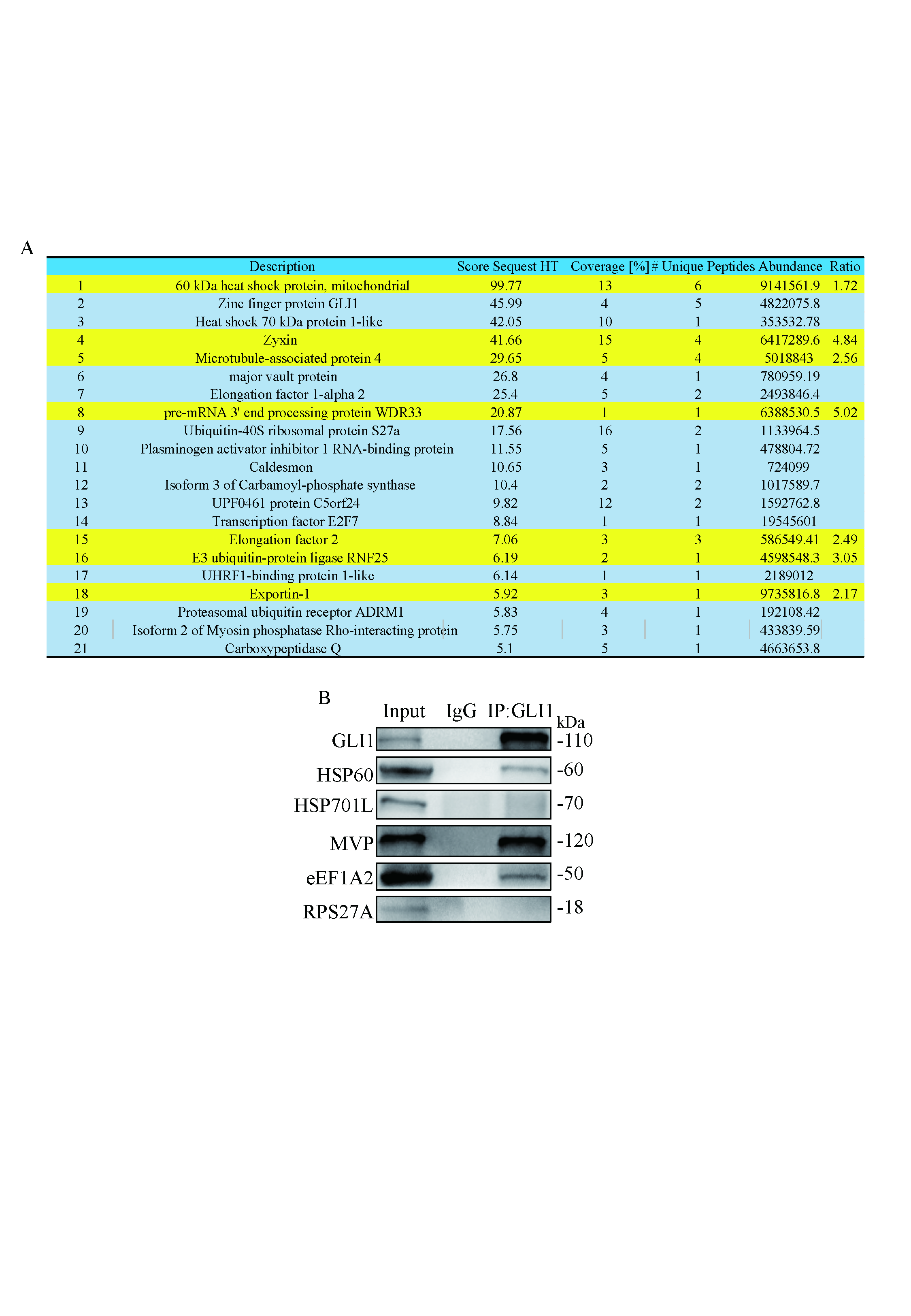

Supplement: Supplementary file 3 — Supplementary Figure 2 [file 41418_2021_749_MOESM3_ESM.tif]

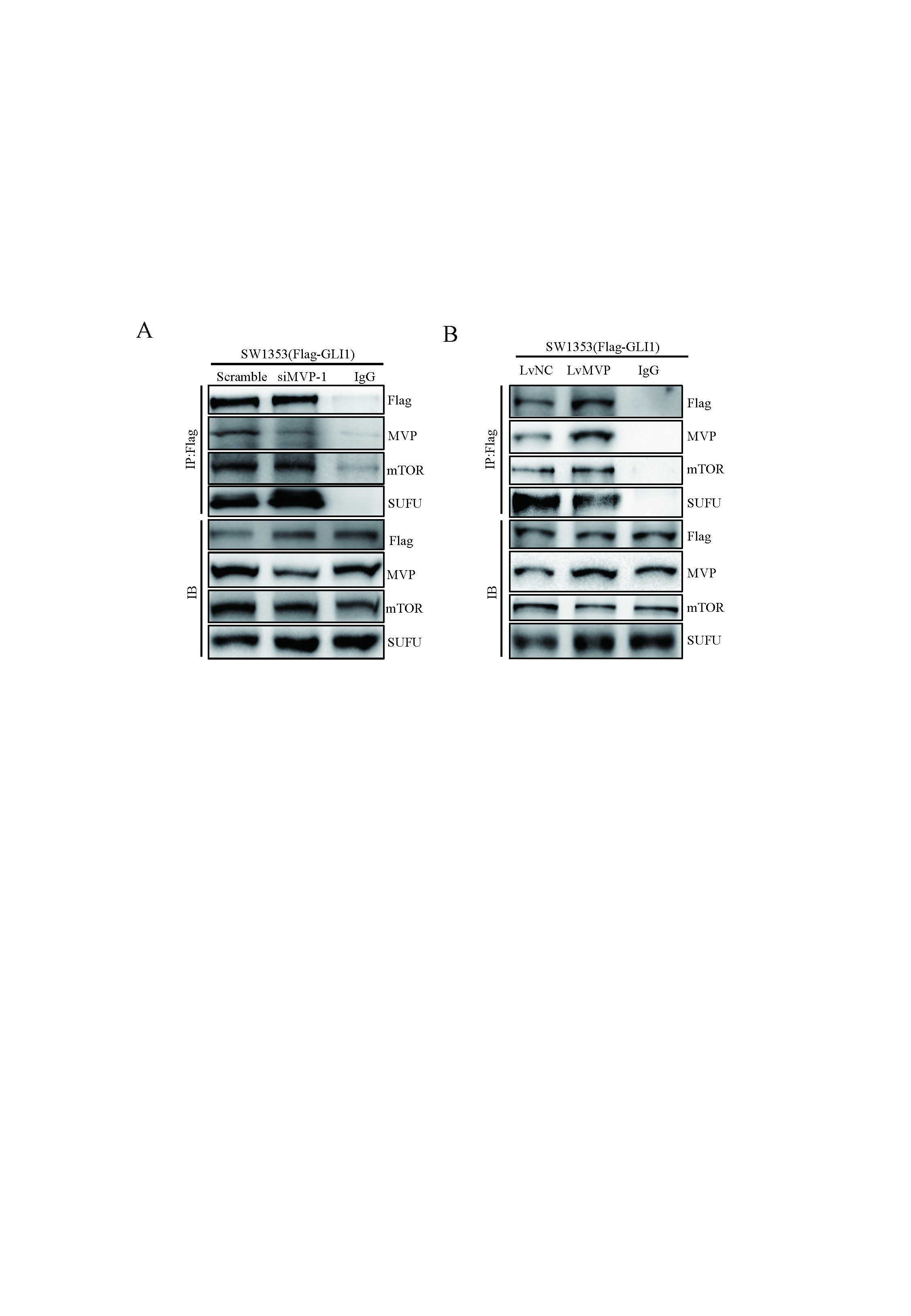

Supplement: Supplementary file 4 — Supplementary Figure 3 [file 41418_2021_749_MOESM4_ESM.tif]

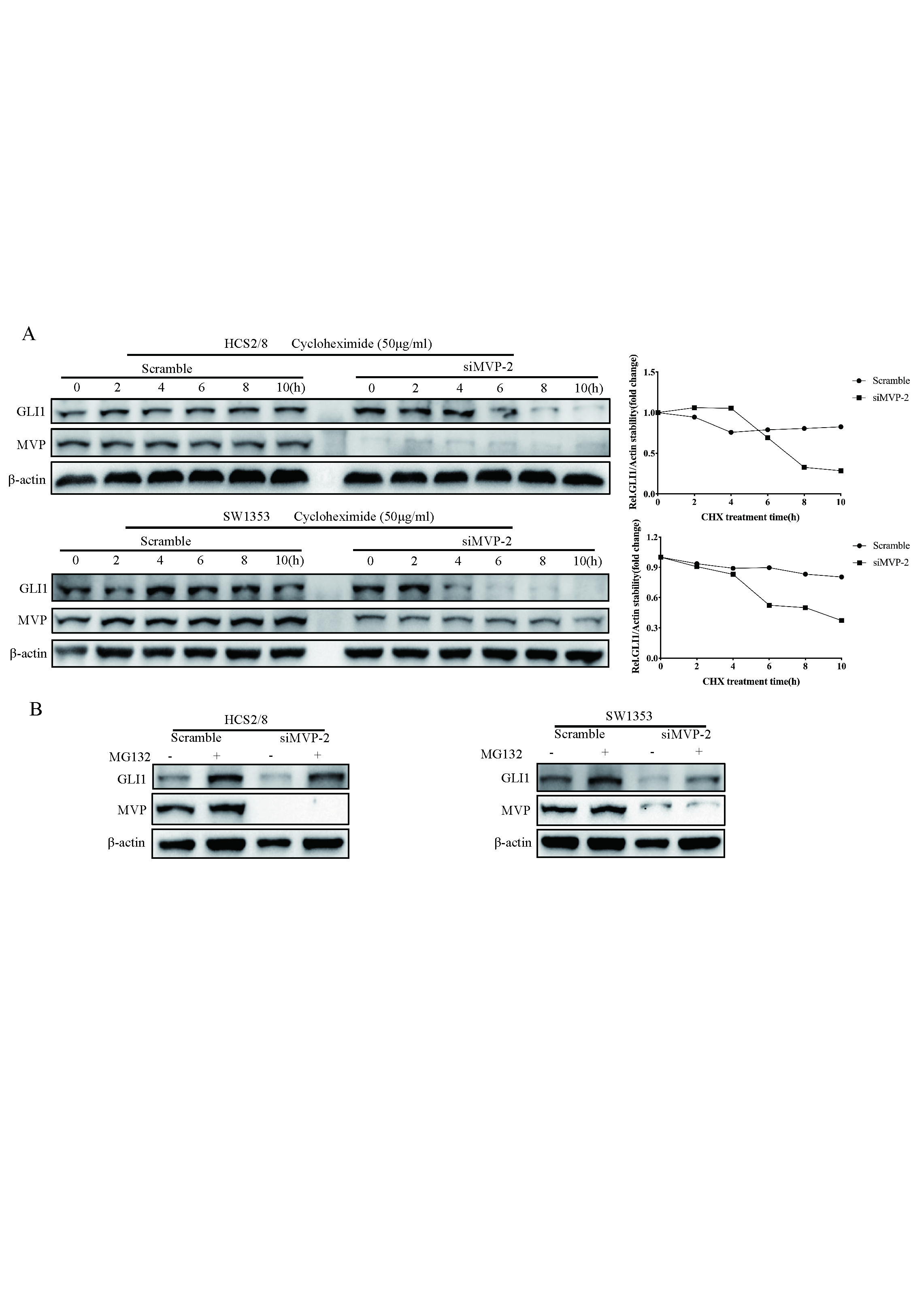

Supplement: Supplementary file 5 — Supplementary Figure 4 [file 41418_2021_749_MOESM5_ESM.tif]

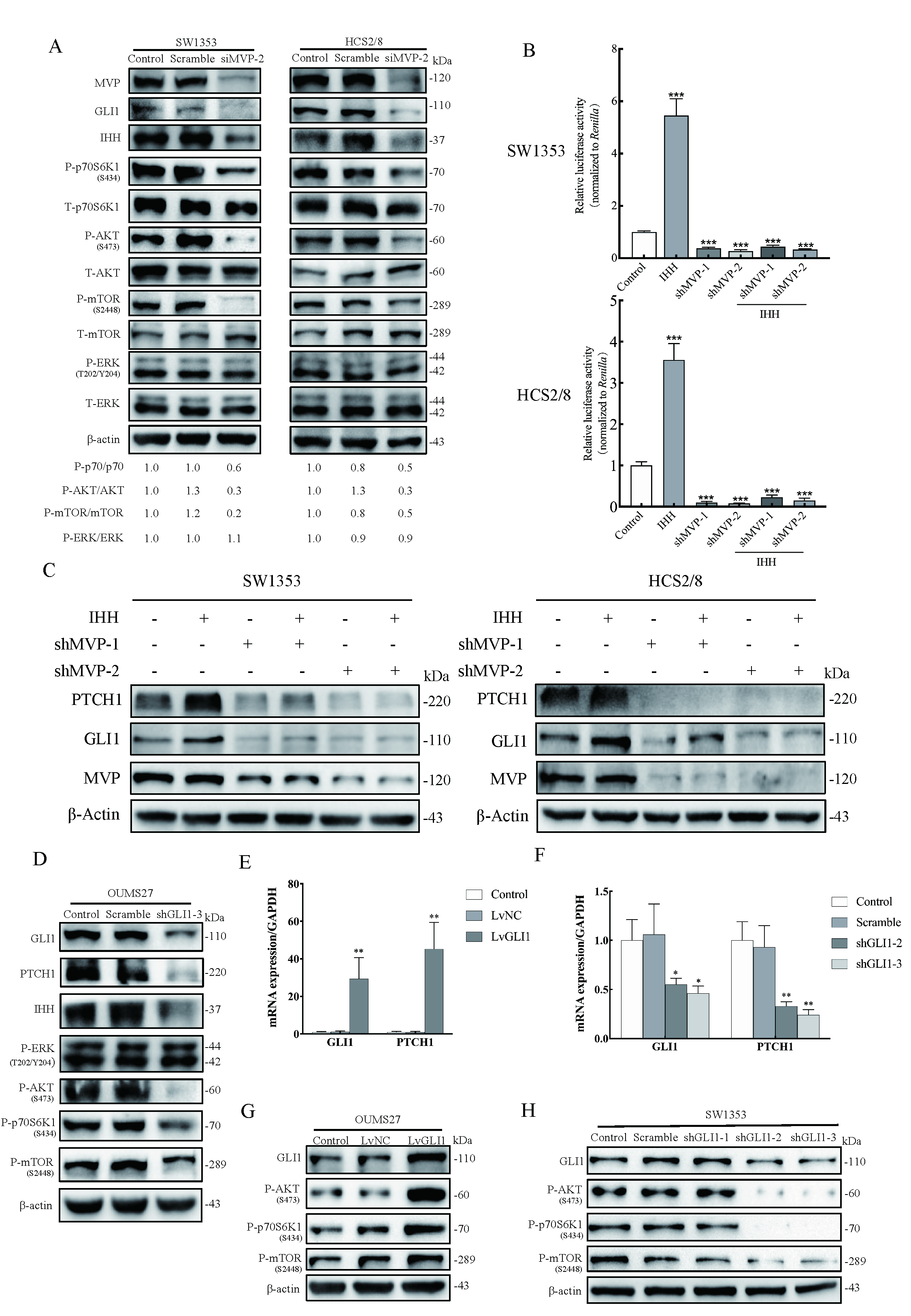

Supplement: Supplementary file 6 — Supplementary Figure 5 [file 41418_2021_749_MOESM6_ESM.tif]

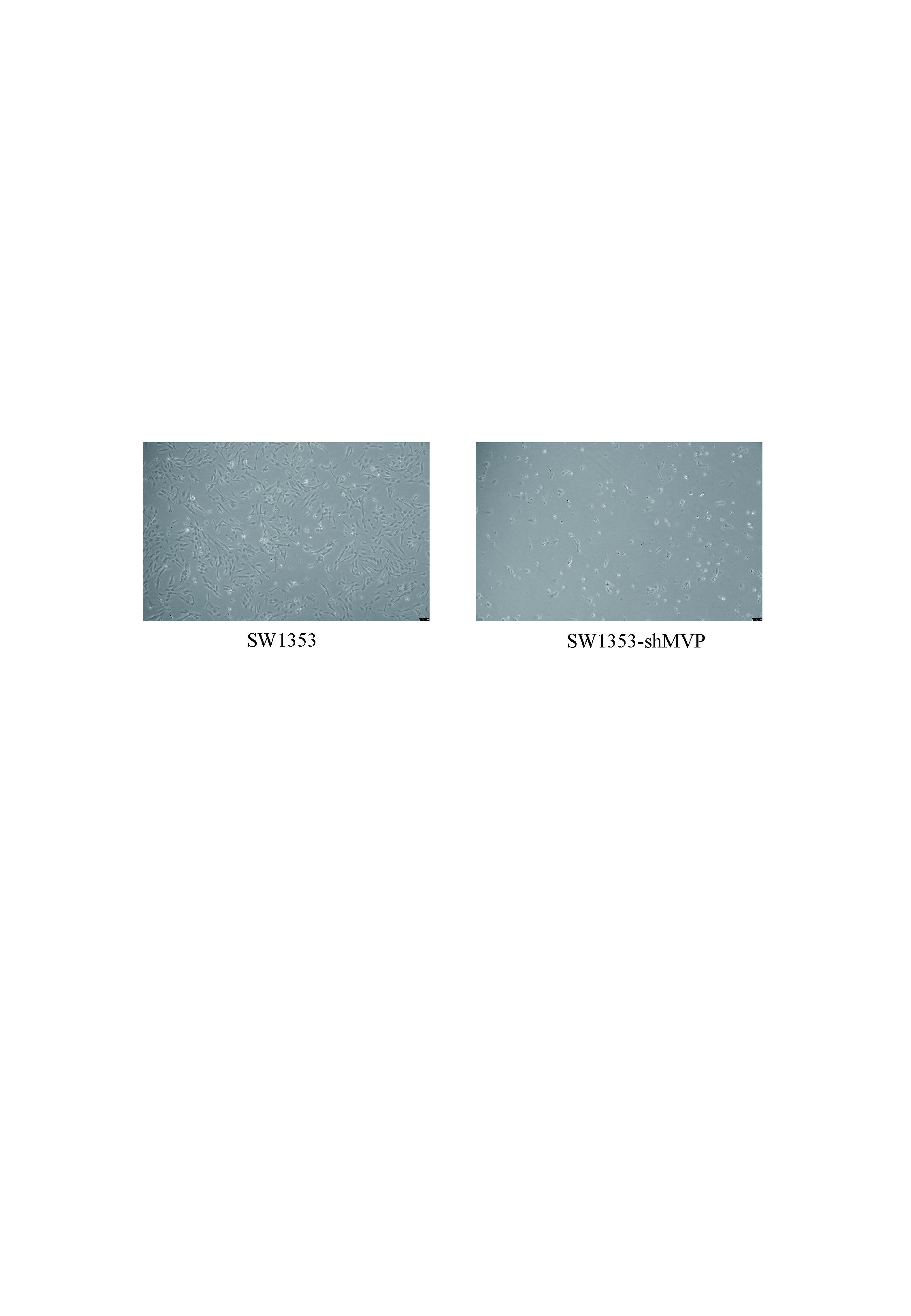

Supplement: Supplementary file 7 — Supplementary Figure 6 [file 41418_2021_749_MOESM7_ESM.tif]

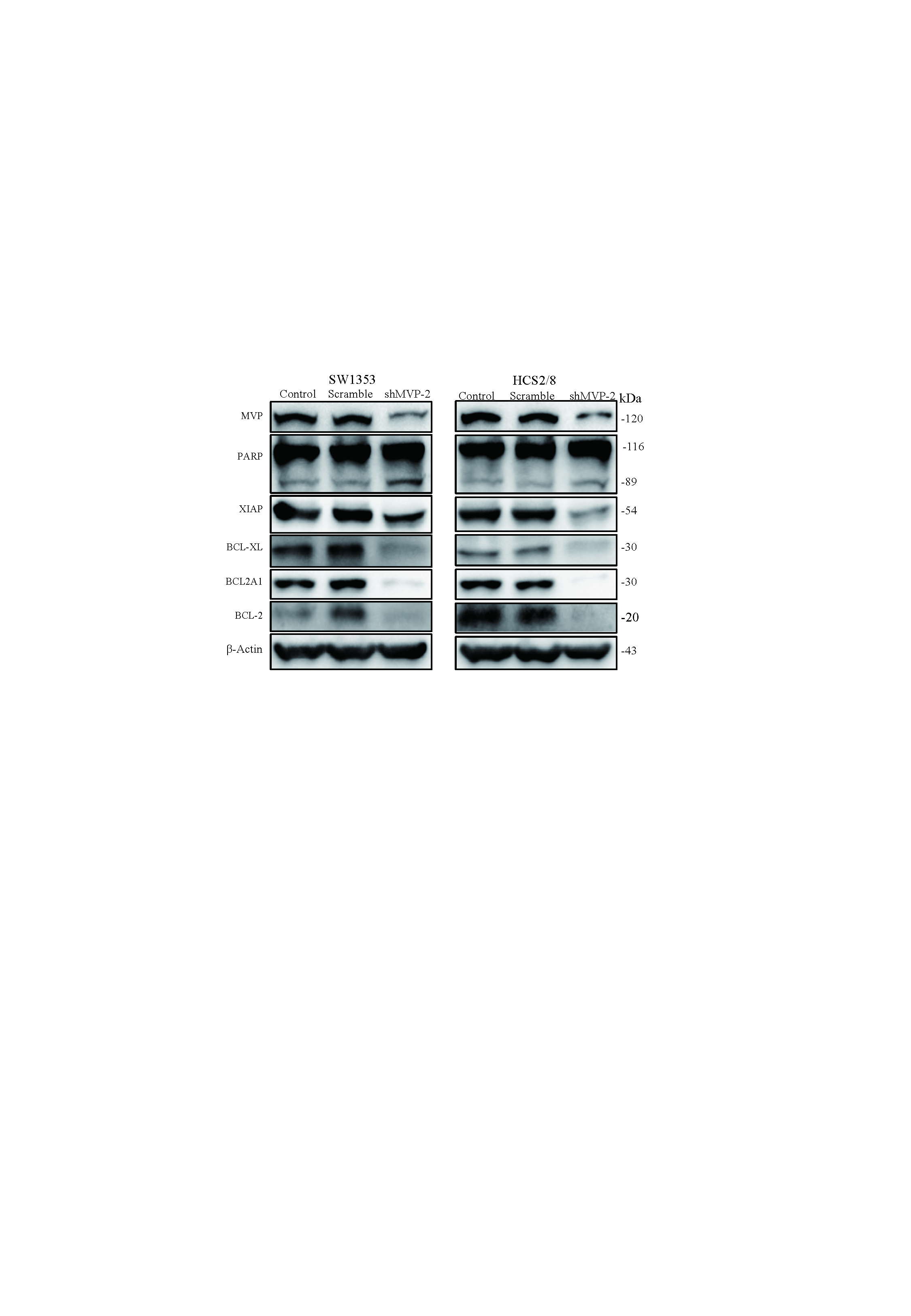

Supplement: Supplementary file 8 — Supplementary Figure 7 [file 41418_2021_749_MOESM8_ESM.tif]

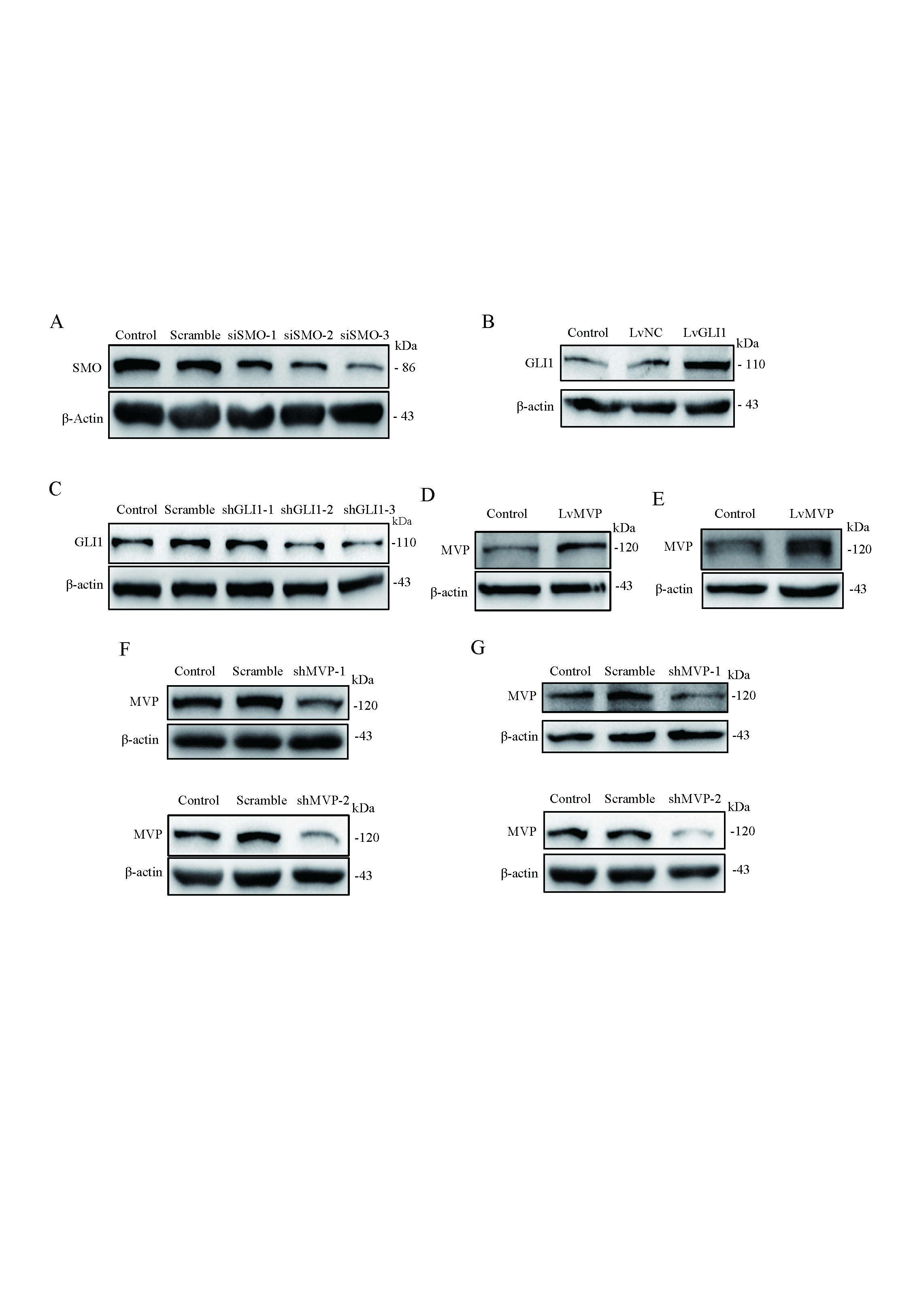

Supplement: Supplementary file 9 — Supplementary Figure 8 [file 41418_2021_749_MOESM9_ESM.tif]
