# Supplementary material for: Constitutive GLI1 expression in chondrosarcoma is regulated by major vault protein via mTOR/S6K1 signaling cascade
Source: Cell Death Differ. 2021 Feb 26;28(7):2221–37. doi: 10.1038/s41418-021-00749-4 (PMC8257592; doi:10.1038/s41418-021-00749-4)
Supplement: Supplementary file 10 — Supplementary information [file 41418_2021_749_MOESM10_ESM.docx]

Supplemental information

Constitutive GLI1 expression in chondrosarcoma is regulated by major vault protein via mTOR/S6K1 signaling cascade

Wei Wang, Taiqiang Yan,^*^ Wei Guo,^*^ Jianfang Niu, Zhiqing Zhao, Kunkun Sun, Hongliang Zhang, Yiyang Yu, Tingting Ren

**Supplementary Figure Legends**

**Supplementary Figure 1.** **(A)** Photographs depicting the morphology of human Primary chondrocyte and chondrosarcoma cell line SW1353. **(B)** Western blot analysis of hedgehog pathway components in SW1353 after SMO over-expression. **(C)** SW1353 cells were treated with cyclopamine (20μM) or SMO gene knockdown, and chromatin was immunoprecipitated with the antibodies of GLI1 and isotype control IgG. Eluted DNA was PCR amplified using primers encompassing the GLI-binding site of the *PTCH1-*promoter or the *GAPDH* coding region (negative control**).**

**Supplementary Figure 2. Identification of proteins interacted with GLI1 in CS cell line SW1353. (A)** Protein profiling results obtained by IP GLI1 combined with LC-MS/MS in SW1353. Inclusion criteria: Score Sequest HT>5; Unique peptide ≥1. Though proteins marked yellow are both detected in IgG and IP group, IP/IgG>1.5 of Score Sequest HT is also considered significant. **(B)** Western blot analysis of the indicated proteins that may interact with GLI1.

**Supplementary Figure 3.** Chondrosarcoma cell line SW1353 were stably transfected with 3xFlag-GLI1, followed by MVP knockdown **(A)** with siRNA targeting MVP and overexpression **(B)** with lentiviral vectors that express full length human MVP. Cells were lysed and subjected to IP with Flag antibody to examine the interaction between GLI1 and indicated proteins. Normal IgG was used as control.

**Supplementary Figure 4. MVP promotes protein stability of GLI1 in chondrosarcoma. (A)** SW1353 and HCS2/8 cells were treated with cycloheximide (CHX, 50μg/ml) for the indicated times, and cell lysates were analyzed by western blot with the indicated antibody. β-actin was used as the loading control, quantification results are shown in the right panel. **(B)** SW1353 and HCS2/8 cells were pre-treated with DMSO or MG132 (10μM) for 8 h, followed by parallel co-treatment with siMVP for 24 h. Cells were lysed and analyzed by western blot with indicated antibodies.

**Supplementary Figure 5. (A)** Western blot analysis of principle proteins involved in IHH, mTOR and MAPK signaling pathway in normal and CS cells with MVP knock-down. Densitometry analysis was performed to quantify the relative phosphorylation levels of p70S6K1, AKT, mTOR and ERK based on the blots. **(B)** Stable MVP gene knockdown cell clones were constructed, and the luciferase activity was detected after 24h treatment with 100 ng/ml IHH in SW1353 and HCS2/8. **(C)** Western blot analysis of the expression of PTCH1 and GLI1 with MVP knock-down and/or IHH (100ng/ml) stimulation. **(D)** Western blot results showing the expression of indicated proteins in OUMS27 cells with GLI1 knockdown. **(E)** Real time RT-PCR results showing the expression of GLI1 and PTCH1 in SW1353 cells over-expressing GLI1. Error bars represent SD (n=6). **(F)** Real time RT-PCR results showing the expression of GLI1 and PTCH1 in OUMS27 cells with GLI1 knock-down. Error bars represent SD (n=6). **(G)** Western blot analysis of the mTOR pathway activation status in OUMS27 with GLI1 overexpression. **(H)** Western blot analysis of the mTOR pathway activation status in SW1353 with GLI1 knockdown. All data are presented as mean ± SD. (*p<0.05, **p<0.01, by Student’s t-test).

**Supplementary Figure 6.** The morphology of SW1353-shMVP cells differed from the parental cell line SW1353. Cells stopped growing after knocking down MVP.

**Supplementary Figure 7.** Western blot analysis of apoptosis related proteins in shMVP transfected and control cells.

**Supplementary Figure 8. Western blot analysis of the overexpression and knockdown efficiency.**

1. Western blot analysis of the knockdown efficiency of siSMO in SW1353.
2. Western blot analysis of the overexpressed GLI1 in SW1353.
3. Western blot analysis of the knockdown efficiency of shGLI1 in SW1353.
4. Western blot analysis of the overexpressed MVP in HCS2/8.
5. Western blot analysis of the overexpressed MVP in OUMS27.
6. Western blot analysis of the knocked down MVP expression in SW1353.
7. Western blot analysis of the knocked down MVP expression in CS-OKB.
